# Manipulating dynamic covalent bonds through direct photoisomerization

**DOI:** 10.1039/d5sc06704a

**Published:** 2025-11-06

**Authors:** Neil D. Dolinski, Alex E. Crolais, Nicholas R. Boynton, Chuqiao Chen, Juan J. de Pablo, Scott A. Snyder, Stuart J. Rowan

**Affiliations:** a Pritzker School of Molecular Engineering, University of Chicago Chicago Illinois 60637 USA stuartrowan@uchicago.edu n.dolinski@columbia.edu; b Department of Chemistry, University of Chicago Chicago Illinois 60637 USA sasnyder@uchicago.edu; c Department of Chemical and Biomolecular Engineering, New York University Brooklyn NY 11201 USA; d Department of Computer Science, New York University NY 10012 USA; e Department of Physics, New York University NY 10012 USA

## Abstract

The wide availability, ease of manipulation, and access to spatiotemporal control make light an attractive stimulus for controlling dynamic covalent chemistries/networks. In this work, a series of photo-isomerizable benzalisoxazolone (BIOx) thia-Michael (tM) acceptors were developed that exhibit an increase in thiol-bonding during irradiation with visible light (455–470 nm). *In situ* photo-NMR experiments demonstrate significant increases in the overall system *K*_eq_ (extent of bonding) for a variety of electronically-substituted BIOx tM acceptors. A combination of experimental and computational studies show that steric interactions between the β-phenyl ring and substituent on the isoxazolone in the *E*-isomer serve as a driving force for the increased *K*_eq_. In addition, it is demonstrated that the power/intensity of the light can be used to tune the system's response. Incorporation of these photoisomerizable motifs into dynamic polymer networks gives access to organogels that exhibit reversible, on-demand, light-triggered stiffening.

## Abstract 2

Photoisomerization-induced destabilization is leveraged to prepare tunable, dynamic Michael acceptors whose *K*_eq_ can be increased upon exposure to light. Incorporation of these acceptors into polymer networks results in photoresponsive dynamic gels.

## Graphical abstract

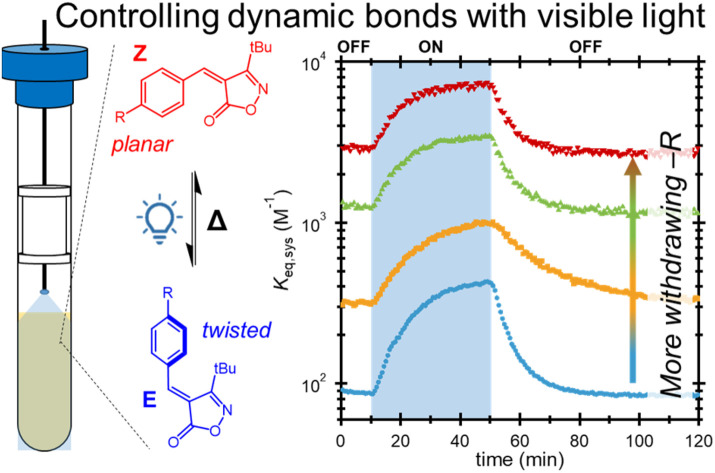

## Introduction

By virtue of their reversible and stimulus-responsive nature,^1^ dynamic covalent chemistries (DCCs) have seen widespread use in applications ranging from drug delivery systems,^2,3^ adhesives,^4,5^ (re)processability/recyclability,^6,7^ and smart/adaptive materials.^8^ The wide suite of currently available DCCs include reversible Diels–Alder reactions,^9–11^ disulfide/diselenide exchange,^12,13^ and transesterifications,^14,15^ to name just a few.^16–19^ Available DCCs cover a wide range of bonding partners with varying characteristics such as exchange mechanism, equilibrium constants, exchange rate(s), catalyst requirements, and the nature of the external stimulus (*e.g.*, heat, pH, light, *etc.*) required to induce a response.^20,21^ The choice of which dynamic covalent bond to incorporate into a polymeric system is critical, as this will determine key properties of the material.

**Fig. 1:**
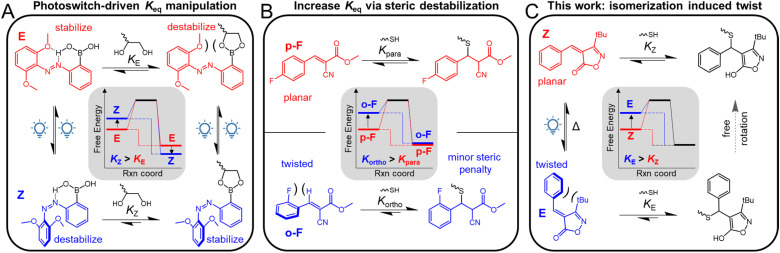
(A) Photoswitchable azobenzene boronic acid/ester system demonstrating the separation of the photoswitch and the dynamic covalent bond. (B) Increased thiol-bonding of *ortho*-substituted BCA acceptors compared to *para*-substituted BCA acceptors on account of destabilization of the free acceptor. (C) Proposed photoswitchable isoxazolone system with a destabilized free acceptor upon photoisomerization.

Light has several attractive features over other stimuli, including the ability to modulate wavelength and intensity, and access to spatiotemporal control. Photoswitches (PSs), a privileged class of compounds that undergo (bi)directional switching in response to light, can undergo a variety of transformations ranging from changes in molecular shape (*e.g.*, *E*/*Z* photoisomerization), conjugation, charge, or even p*K*_a_.^22^ Recent studies have elegantly demonstrated that coupling PS groups (*e.g.*, azobenzenes,^23–25^ hydrazones,^26,27^ spiropyrans,^28^ and diarylethenes^29,30^) in close proximity to a dynamic covalent bond allows for manipulation of bond exchange. Often the PS moiety is not directly involved in the dynamic bonding itself, but modulates the dynamic bond through secondary interactions. An elegant example of this is a photoswitchable azobenzene–boronic ester system developed by the Kalow group that leverages the photoisomerization of azobenzene (from *trans* to *cis*) to result in an increase in diol bonding due to destabilization of the unbonded *cis* boronic acid and stabilization of the bonded ester relative to the *trans* isomer (Fig. 1A).^24^ Given the utility of such systems it is of interest to explore the development of synthetically-simple, tunable, light-responsive dynamic bonds.

In recent years, thia-Michael (tM) chemistry, the conjugate addition of a thiol to an activated alkene, has seen exciting applications beyond its traditional use of forming a static C–S bond.^31^ Through tailoring the substituents on the activated alkene, the dynamic behavior of the tM bond can be readily tuned, leading to a range of reported dynamic tM polymer networks.^32–34^ In many of these systems, elevated temperatures and/or the presence of base are required to access dynamic exchange. There is an interesting subset of tM acceptors, such as benzalcyanoacetate (BCA),^35^ benzalcyanoacetamide (BCAm),^36^ and benzalisoxazolone (BIOx),^37^ which can undergo catalyst-free, room-temperature thiol exchange. The exploration of these systems has helped spearhead investigations into the use of room temperature dynamic bonds in materials with applications including hydrogels,^38–40^ colloidal systems,^41–43^ and pluripotent materials.^44^ Importantly, the equilibrium constant (*K*_eq_) of these acceptors, and thus the thermomechanical properties of their derived materials, can modularly be tuned through electronic modifications of their β-phenyl rings.^45^ Recently, it has been demonstrated that *ortho* substitution of the β-phenyl ring can be leveraged to significantly enhance the *K*_eq_ of these tM acceptors.^46^ Careful selection of *ortho* substituents allows for the destabilization of the otherwise planar tM acceptor through twisting of the β-phenyl ring, while only minorly destabilizing the thiol-adduct. This results in an overall net increase in Δ*G*, and therefore *K*_eq_ (Fig. 1B).

Reported herein is an investigation that builds on these prior studies by exploring if photoisomerization of a sterically twisted isomer would allow light to be used to increase the *K*_eq_ of a dynamic tM system on demand. Specifically, the study focuses on how the *Z*-to-*E* photoisomerization of sterically hindered BIOx acceptors can be used to modulate the equilibrium of dynamic tM reactions (Fig. 1C). The synthesis of such BIOx acceptors is both facile and modular, which allows access to derivatives with a range of electronic and steric modifications. Importantly, this demonstration of sterics-focused molecular design has the potential to introduce photo-responsive behaviors across other DCC motifs, simplifying synthesis and offering new design handles for researchers.

## Results and discussion

**Fig. 2:**
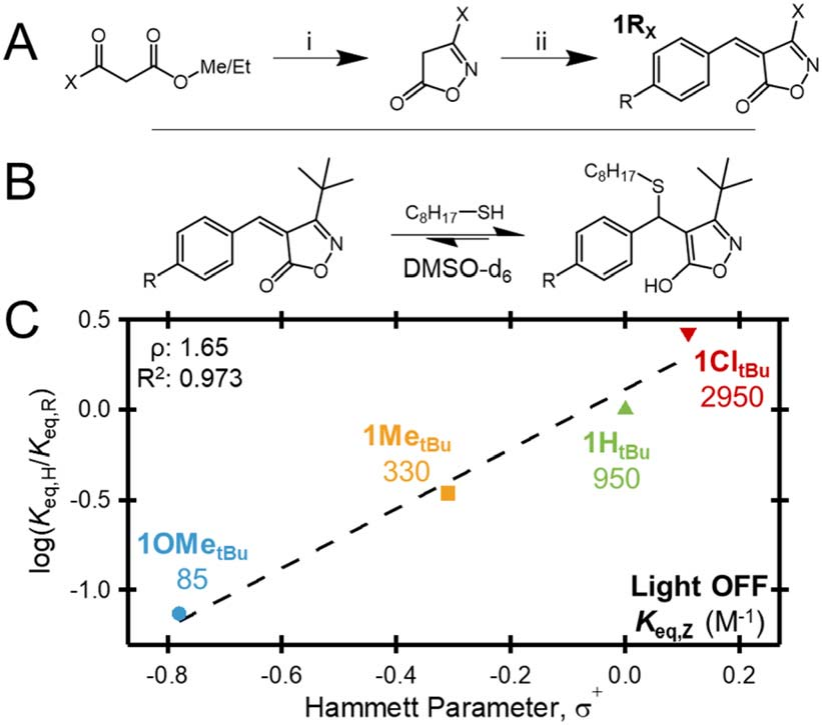
(A) Synthesis of 1R_x_ acceptors: (i) ketoester, hydroxylamine hydrochloride, NaOAc, ethanol, 50 °C; (ii) isoxazolone, benzaldehyde, piperidine, isopropanol, 50 °C. (B) Equilibrium between 1R*_t_*_Bu_ acceptors and OT. (C) Hammett plot of *Z* isomers of 1R*_t_*_Bu_ acceptors *versus* the *σ*^+^ Hammett parameter of R.

Synthesis of the targeted acceptors can be accomplished in two steps from readily available starting materials: (i) a ring closure between the appropriate alkyl (–X) keto ester and hydroxylamine followed by (ii) a Knoevenagel condensation with the appropriate substituted (–R) benzaldehyde (Fig. 2A) (see the SI for full synthetic details). Throughout this work, the prepared acceptors are denoted as 1R_X_, where R represents the substituent on the β-phenyl ring and X is the bulky alkyl group at the 3-position of the isoxazolone. A signature feature of the BCA, BCAm, or BIOx-based tM acceptors is their ability to tune equilibrium and exchange kinetics through the addition of electronic substituents to the β-phenyl ring.^45^ To explore if this important characteristic is maintained, a series of 1R*_t_*_Bu_ acceptors were prepared with a range of electronic substituents (–OMe, –Me, –H, and –Cl) at the –R position. Gratifyingly, in agreement with previous findings, the measured (dark) equilibrium constants (*K*_eq,*Z*_) were found to have a linear relationship with *σ*^+^ Hammett parameters,^37,45^ with *K*_eq,*Z*_ values ranging from 85 to 2950 M^−1^ for 1OMe*_t_*_Bu_ and 1Cl*_t_*_Bu_, respectively (Fig. 2C). Furthermore, the determined slope (*ρ*) of 1.65 is intermediate between those of previously reported BIOx (2.08 (ref. 37)) and BCA (1.37 (ref. 45)) systems.

To determine ideal wavelengths to irradiate the suite of 1R_X_ acceptors, UV-vis studies were carried out over a range of concentrations in DMSO (Fig. S1–S3). It was determined that blue light, either 455 or 470 nm depending on the –R modification (only R = OMe requiring 470 nm), would be most suitable for further study. Indeed, upon exposure to these wavelengths, new *E*-isomer peaks were readily observed *via* NMR and were found to rapidly disappear when the light was turned off (Fig. S4).

As an initial investigation into the impact of photoisomerization on the reaction equilibrium, a 100 mM equimolar mixture of 1H*_t_*_Bu_ and 1-octanethiol (OT) was prepared and allowed to equilibrate in the dark for 12 hours prior to evaluation. During irradiation, the planar, energetically favorable *Z*-isomer undergoes isomerization to the twisted higher energy *E*-isomer.

**Fig. 3:**
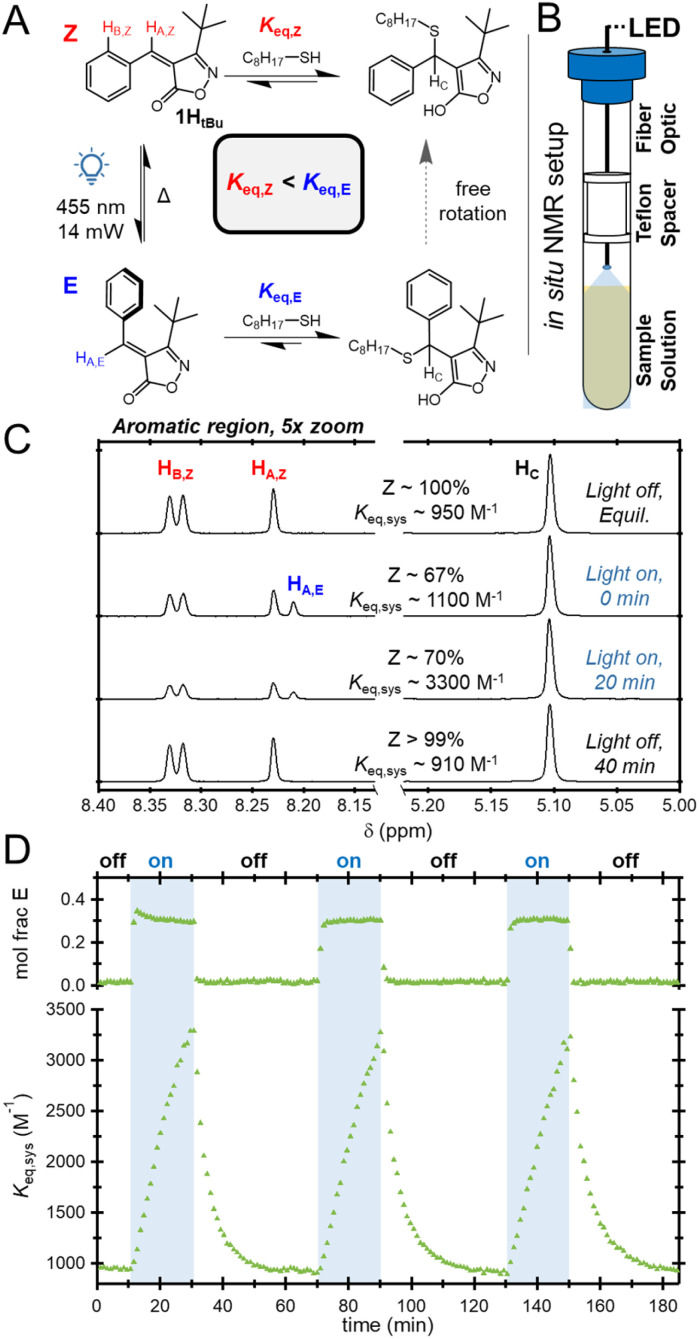
(A) Equilibria involved in thiol-bonding and photoisomerization. (B) Diagram of the *in situ* LED NMR setup. (C) ^1^H NMR spectra of 1H*_t_*_Bu_ equilibrated with OT without and during irradiation with 455 nm light. (D) Cycling experiment (455 nm irradiation for 20 min followed by a 40 min dark interval) for 1H*_t_*_Bu_ equilibrated with OT.

This destabilization of the unbonded state should, in turn, shift the equilibrium of the reaction to the bonded state (Fig. 3A). Importantly, the bonded state is free to rotate, and should rapidly revert to its lowest energy configuration (in line with the *Z* isomer), allowing the system to return to the initial equilibrium (*K*_eq,*Z*_) after the light is turned off. To monitor this process in real time, a previously reported fiber-coupled NMR technique was adopted, as schematically shown in Fig. 3B.^47^

In agreement with expectations, the NMR spectrum of the equilibrated 1H*_t_*_Bu_ sample (dark) was found to have only peaks related to the unbound *Z* isomer (H_A,*Z*_ @ 8.23 ppm and H_B,*Z*_ @ 8.32 ppm) and the corresponding thiol adduct (H_C_ @ 5.10 ppm), Fig. 3C. However, upon irradiating the sample with 455 nm light (14 mW), rapid *Z* to *E* isomerization was observed, yielding a new characteristic peak at 8.21 ppm (H_A,*E*_) and an *E* isomer mole fraction of *ca.* 0.3. At this early stage of irradiation, a small increase in the fraction of thiol adducts was measured, described most conveniently by the overall system equilibrium constant, *K*_eq,sys_ (not distinguishing between isomers), as defined below.
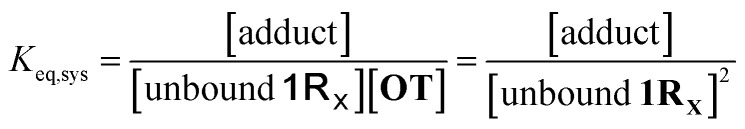


For the 1R*_t_*_Bu_ system *K*_eq,sys_ = *K*_eq,*Z*_ in the dark as 1R*_t_*_Bu_ exists as the 100% *Z* isomer. Irradiation with light results in the appearance of a peak in the NMR consistent with the formation of the *E* isomer, along with a slight increase in *K*_eq,sys_. An additional 20 minutes of irradiation was found to significantly increase *K*_eq,sys_ from its initial (dark) value of 950 to 3300 M^−1^, all while maintaining a consistent mole fraction of the *E* isomer. At this point, irradiation was ceased and the sample was allowed to equilibrate for 40 minutes. As expected, without irradiation, *E* isomer peaks vanished and the system returned to its initial dark equilibrium position.

To further investigate the performance of this system, the experiment was repeated under cycling conditions with continuous monitoring under the same irradiation conditions (455 nm, 14 mW), Fig. 3D. As before, upon irradiation the fraction of *E* isomer rapidly increased to *ca.* 30%, while the *K*_eq,sys_ was found to gradually increase with exposure time. Upon turning the light off, *E* isomer peaks rapidly vanished and the *K*_eq,sys_ returned back to the initial value. Importantly, these features were found to be consistent across cycles, implying that this strategy for manipulating dynamic bonds is reversible.

**Fig. 4:**
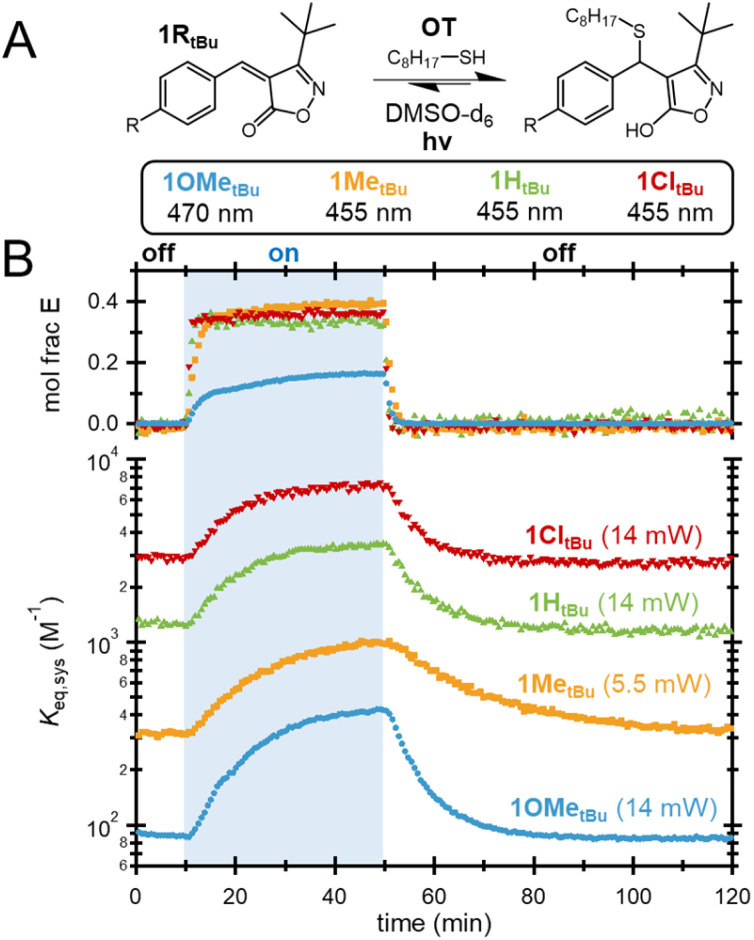
(A) Equilibrium between 1R*_t_*_Bu_ and OT. (B) Mole fraction of the *Z* isomer and overall system *K*_eq_ (*K*_eq,sys_) of 1R*_t_*_Bu_ acceptors equilibrated with OT during the NMR photoisomerization experiment.

With the ability to control bonding through light demonstrated using 1H*_t_*_Bu_, attention was next turned to other electronically-modified derivatives, 1R*_t_*_Bu_ (Fig. 4A). As the dark *K*_eq,*Z*_ for the different derivatives spans a wide range, a fixed concentration of 40 mM was chosen to optimize the NMR signal and penetration depth across species. Importantly, all 1R*_t_*_Bu_ derivatives were found to experience significant shifts in bonding upon irradiation with the appropriate wavelength. Notably, for all but 1OMe*_t_*_Bu_, irradiation to a consistent mole fraction of 0.30–0.35 *E* isomers drives a 3-fold increase in *K*_eq,sys_ from the initial *K*_eq,*Z*_, returning to the initial equilibrium state after irradiation is ceased. In the case of 1OMe*_t_*_Bu_, an impressive 5× shift in *K*_eq,sys_ was measured, despite a much smaller fraction of *E* isomer (*ca.* 0.15). This lower extent of isomerization is partially attributed to limited penetration depth on account of the low *K*_eq,*Z*_ yielding a higher concentration of absorbing unbound species.

**Fig. 5:**
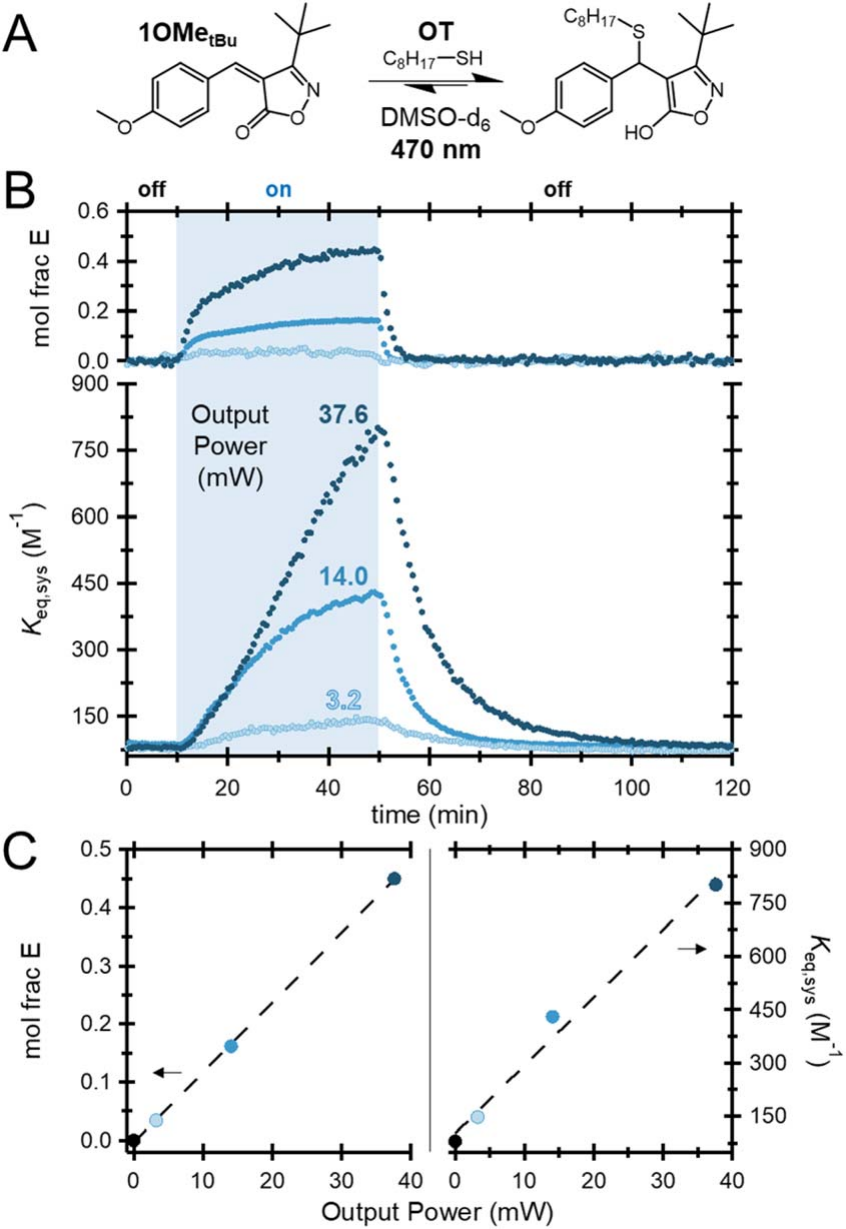
(A) Equilibrium between 1OMe*_t_*_Bu_ and OT. (B) Mole fraction of the *Z* isomer and overall system *K*_eq_ (*K*_eq,sys_) of 1OMe*_t_*_Bu_ equilibrated with OT during the NMR photoisomerization experiment at varying LED output powers (3.2 to 37.6 mW). (C) Mole fraction of *Z* isomer and maximum system *K*_eq_*versus* LED output power.

Notably, during the previous suite of measurements, it was found that significantly less LED power was required to drive 1Me*_t_*_Bu_ to a similar mole fraction of *E* isomer as the other species sensitive to 455 nm light (5.5 *vs.* 14 mW). As such, a series of experiments with varied LED output power was carried out on 1OMe*_t_*_Bu_ (Fig. 5A and B) and 1Me*_t_*_Bu_ (Fig. S9). Intuitively, increased LED power was found to increase the *E* isomer population, in turn driving larger increases in *K*_eq,sys_. Pleasingly, for both species, the isomer fraction and the maximum *K*_eq,sys_ were found to be effectively linear with respect to LED power under the measured conditions, Fig. 5C. This feature is beneficial as it can serve as an additional tuning handle for the predictable manipulation of bonding.

**Fig. 6:**
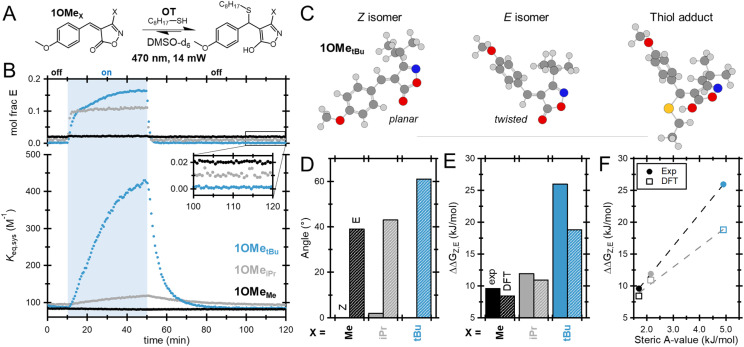
(A) Equilibrium between 1OMe_X_ acceptors and OT. (B) Mole fraction of the *Z* isomer and overall system *K*_eq_ (*K*_eq,sys_) of 1OMe_X_ equilibrated with OT during the NMR photoisomerization experiment. (C) Conformations of *Z* and *E* isomers as well as the thiol-adduct of 1OMe*_t_*_Bu_. (D) Dihedral angles between the β-phenyl ring and α,β-unsaturated bond of *Z* (solid) and *E* (striped) isomers of 1OMe_X_ acceptors. (E) ΔΔ*G Z* and *E* isomers of 1OMe_X_ acceptors and determined from experimental values (solid) and computations (striped). (F) ΔΔ*G* of *Z* and *E* isomers of 1OMe_X_ acceptors *versus* steric *A*-values of –X.

To develop a better understanding of the underlying thermodynamic driving force, a series of acceptors with varied steric demand 1OMe_X_ (–X = –Me, –iPr, and –*t*Bu) were investigated *via* a combination of experiments and simulations. Equilibrated mixtures of 1OMe_X_ and OT were investigated under fixed 14 mW irradiation conditions (Fig. 6A). The less hindered 1OMe_iPr_ was found to undergo comparable *Z* to *E* isomerization to 1OMe*_t_*_Bu_ during exposure; however the overall *K*_eq,sys_ was only minorly impacted, rising from *ca.* 90 to 150 M^−1^ (Fig. 6B). The least hindered species, 1OMe_Me_, did not undergo any measurable photoisomerization during irradiation, and was found to exhibit *ca.* 2 mole percent of *E* isomer throughout the experiment (Fig. S10).

To further explore the impact of the X-substituent on the photoisomerization, computational studies on the suite of 1OMe_X_ acceptors were carried out. Density functional theory (DFT) calculations were performed using the Gaussian  16 package with the M06-2X/6-311+G(d,p) level of theory.^48^ The free energy differences were computed in a vacuum and in DMSO with continuum solvent models (see the SI for details).^49^ Representative configurations of *Z* and *E* isomers for the *t*Bu acceptor as well as the thiol adduct are shown in Fig. 6C (the related configurations for 1OMe_Me_ and 1OMe_iPr_ are shown in Fig. S11). For all *Z* isomers, the dihedral angle between the phenyl group and the isoxazolone ring is close to 0 across the series suggesting that added steric bulk at the X position does not impact the stable *Z*-isomer. In contrast, the dihedral angle for the *E*-isomers across the series was found to significantly increase with the size of the alkyl substituent (Fig. 6D). Both simulations and calculations from experimental data found that all *E*-isomers possess higher free energy than the corresponding *Z*-isomers, attributed to steric repulsion between the alkyl (–X) group and the phenyl ring. This difference in free energy between *Z*- and *E*-isomers (ΔΔ*G*_*Z*,*E*_) was found to increase from 9.6 to 26 kJ per mol (experimental) or from 8.4 to 19.2 kJ per mol (DFT) as the –X group increased in steric bulk from –Me to –*t*Bu (Fig. 6E). In agreement with the initial hypothesis, increased steric demand (quantified through steric *A*-values, the Δ*G* value between different conformations of a substituted cyclohexane) results in a linear increase in ΔΔ*G*_*Z*,*E*_, in turn leading to larger changes in *K*_eq,sys_ (Fig. 6F).

**Fig. 7:**
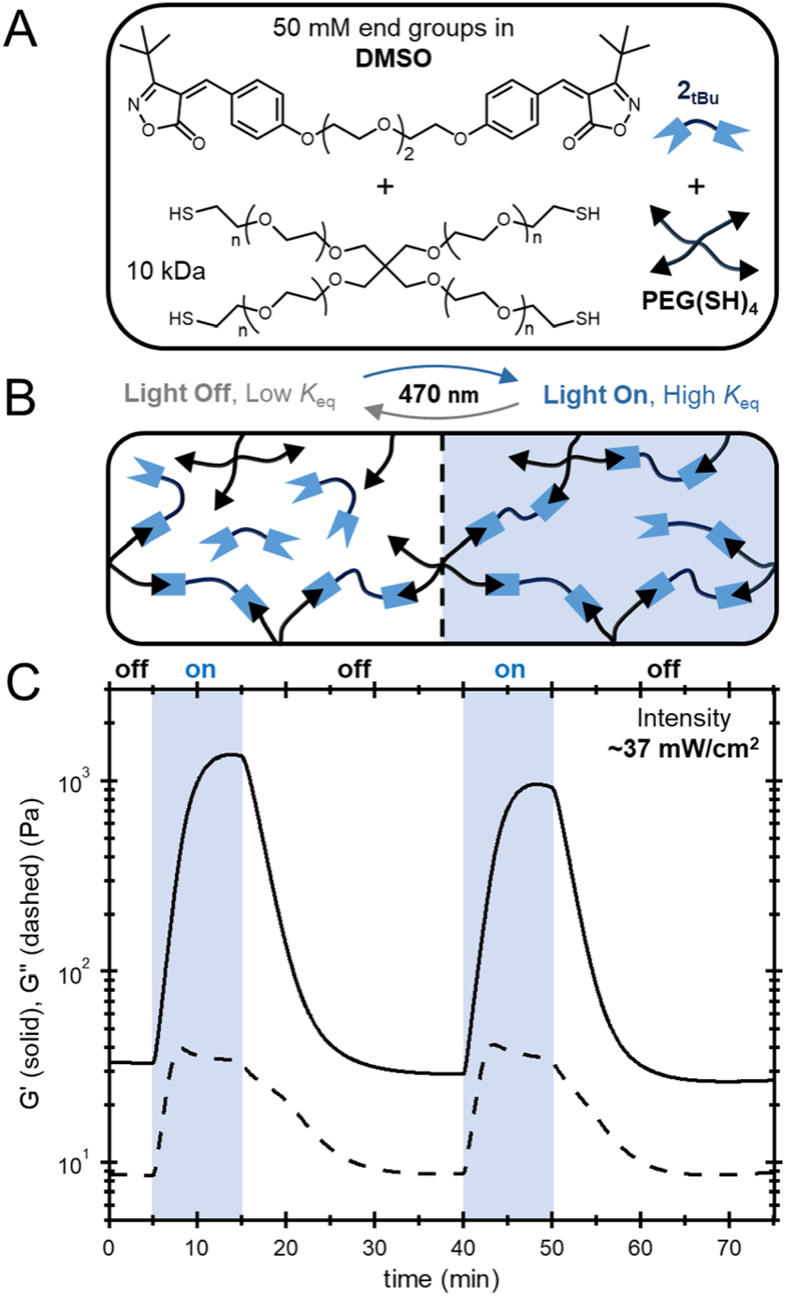
(A) Chemical structures used in the synthesis of the organogel. (B) Diagram demonstrating increase in thiol-bonding and network connectivity upon irradiation. (C) Storage and loss moduli of the organogel during cycles of irradiation (470 nm) and darkness.

Having confirmed that the photoisomerization of these tM acceptors can be used to manipulate *K*_eq,sys_, the next goal was to explore if this effect could also be observed in dynamic polymer networks. Upon exposure to light, the increase in *K*_eq,sys_ would lead to increased crosslink density and modulus while dark periods would restore the initial properties, akin to recently reported light-stabilized dynamic materials.^50^ To this end, a dynamic organogel was targeted. As the largest light-induced changes in *K*_eq_ were measured in the 1OMe*_t_*_Bu_ system, a ditopic species, 2*_t_*_Bu_, was prepared by connecting through ether bonds, electronically similar to methoxy –R groups. With the ditopic monomer in hand, organogel samples were prepared by mixing 2*_t_*_Bu_ and a thiol-terminated 4-arm poly(ethylene glycol) (PEG(SH)_4_, *M*_n_ = 10 kDa) macromonomer in DMSO (targeting 50 mM end group concentration). After mixing, the solution was equilibrated in the dark overnight, yielding a soft gel (Fig. 7A and B). The 50 mM end group concentration was chosen to yield a bonded fraction of *ca.* 0.61 (assuming a *K*_eq,*Z*_ of 85 M^−1^), close to the predicted critical gelation conversion by Flory–Stockmayer theory (*ca.* 0.58). After formation, the sample was loaded into a rheometer equipped with a 470 nm collimated LED and its mechanical properties were evaluated using small amplitude oscillatory shear rheology (setup shown in Fig. S12). Prior to irradiation, the sample exhibited a storage modulus of just over 30 Pa. Gratifyingly, exposure to 470 nm light was found to significantly increase the modulus of the sample, which plateaued at just over 1.2 kPa (Fig. 7C). Upon turning the light off, the sample was subjected to an additional on/off cycle, producing nearly identical results, with a *ca.* 40-fold increase in *G*′ during light exposure and subsequent return to equilibrium after the light was turned off.

## Conclusions

In summary, a new dynamic tM acceptor platform with photo-controllable *K*_eq_ has been developed using a molecular design in which both the photoswitchable and dynamic chemistry moieties are one and the same. Critically, these light sensitive DCC motifs can be readily prepared and derivatized through simple reactions, allowing tuning of the baseline *K*_eq_ and the overall photo-response. This isomerization-driven *K*_eq_ enhancement is tunable through steric bulk on the isoxazolone ring, with larger substituents producing more pronounced changes. In addition, it is shown that incorporation of this motif into organogels allows for impressive stiffening (*ca.* 40× increase in the storage modulus) upon light irradiation. Critically, this direct photoisomerization approach should be amenable to other dynamic bonding motifs, greatly expanding opportunities for applying light-responsive dynamic covalent chemistry.

## Author contributions

Conceptualization: N. D. D., A. E. C, C. C., and S. J. R. Methodology: N. D. D., N. R. B., C. C., and S. J. R. Investigation: N. D. D., A. E. C., N. R. B., and C. C. Visualization: N. D. D. Writing – original draft: N. D. D., A. E. C., and S. J. R. Writing – review and editing: N. D. D., A. E. C., N. R. B., C. C., J. J. dP., S. A. S., and S. J. R. Funding acquisition: S. J. R., S. A. S., and J. J. dP.

## Conflicts of interest

There are no conflicts to declare.

## Supplementary Material

SC-OLF-D5SC06704A-s001

## Data Availability

The data that support the findings of this study are available from the corresponding authors upon reasonable request. Additional data supporting this work can be found in the supplementary information (SI). Supplementary information: experimental procedures, UV-Vis spectra, NMR data, computational details, and rheology data. See DOI: https://doi.org/10.1039/d5sc06704a.
