# Supplementary material for: Manipulating dynamic covalent bonds through direct photoisomerization
Source: Chem Sci. 2025 Nov 6;16(48):23019–25. doi: 10.1039/d5sc06704a (PMC12590959; doi:10.1039/d5sc06704a)
Supplement: SC-016-D5SC06704A-s001 [file SC-016-D5SC06704A-s001.pdf]

Supplementary Information (SI) for Chemical Science.
This journal is © The Royal Society of Chemistry 2025

Electronic Supplementary Information

Manipulating dynamic bond equilibrium through direct photoisomerization

Neil D. Dolinski[‡,*], Alex E. Crolais[‡], Nicholas R. Boynton, Chuqiao Chen,
Juan J. de Pablo, Scott A. Snyder*, Stuart J. Rowan*

Table of Contents

1. EXPERIMENTAL DETAILS S2
- **A. Materials** S2
- **B. Instrumentation** S2
- **C. Synthesis** S3
- **D. Gel Formation** S7

2. CHARACTERIZATION S8
- **A. UV-Vis Spectroscopy** S8
- **B. NMR Analysis** S10
- **C. Computational Details** S15
- **D. Free Energy Calculations** S17
- **E. Shear Rheology** S19

REFERENCES S21

1. EXPERIMENTAL DETAILS

1A. Materials

Methyl acetoacetate (99%), methyl isobutyrylacetate (97%), ethyl 4,4-dimethyl-3-oxovalerate (98%), and 4-chlorobenzaldehyde (>97%) were purchased from TCI Chemicals. Benzaldehyde (99%) p-anisaldehyde (98%), p-tolualdehyde (97%), hydroxylamine hydrochloride (98%), triethylene glycol (99%), and piperidine (99%) were purchased from Sigma Aldrich. 4-hydroxyacetophenone (99%) was purchased from Alfa Aesar. Sodium acetate (≥99%) and potassium carbonate (≥99%) were purchased from Fisher Scientific. All solvents were purchased from Fisher and all deuterated solvents were purchased from Oakwood or Cambridge Isotope Laboratories. All chemicals were used as received unless otherwise noted.

Safety Statement: No chemical or procedure used poses a significant safety hazard when used with proper lab safety protocols.

1B. Instrumentation

Nuclear Magnetic Resonance (NMR): NMR spectroscopy was performed using either a 400 MHz Bruker Avance III HD; Ascend 9.4 Tesla NMR, a 500 MHz Bruker Avance III HD; UltrashieldPlus 11.7 Tesla NMR, or a 600 MHz Bruker Avance Neo; Ascend 14.1 Tesla NMR.

High-Resolution Mass Spectrometry (HRMS): High-resolution mass spectra were recorded on Agilent 6244 Tof-MS using ESI (Electrospray Ionization) at the University of Chicago Mass Spectroscopy Core Facility.

Fiber-coupled NMR: In situ photochemical NMR was carried out using a previously reported approach.[1] Briefly, 455 or 470 nm LEDs (Thorlabs M455F3 or M470F4) were coupled into a multimode optical fiber with a flat end cleave through SMA connection. The optical fiber was fed into the NMR tube through a standard NMR cap with a hole drilled through the center. The fiber was centered above the sample through the use of a custom-machined Teflon spacer. The LED was manually controlled by a manufacturer power supply (Thorlabs LEDD1B T-cube driver). Intensity was measured from the tip of the optical fiber using a Honle UV-meter equipped with a sensor suited for measurement in the range of 280–550 nm. A spectrophotometer (Ocean Flame-S-UV-Vis) was used to measure LED emission profiles.

Shear Rheology: Shear rheology was performed using a TA Instruments Discovery HR-30 shear rheometer equipped with lower optical plate and upper Peltier plate (25 mm) accessories. A 470 nm collimated LED (Thorlabs M470L5-C1) was mounted below the optical plate and focused to irradiate the 25 mm sample area. To minimize the influence of ambient light, the measurement area was covered using aluminum foil after loading the sample. Intensity was estimated as 30% of reported collimated LED output power (via T-cube settings) focused to a 25 mm area, or ~30 mW/cm^2.

1C. Synthesis

General Procedure A: Isoxazolone Synthesis

$HONH_2 \cdot HCl$ (2 equiv.) and NaOAc (2 equiv.) were added to a round bottom flash charged with EtOH and allowed to stir for 5 minutes. After, the β-ketoester (1 equiv.) was added. The reaction was heated to 50 °C and allowed to stir overnight. The solution was then concentrated under reduced pressure and dissolved in CH_2Cl_2. The organic phase was then washed with a concentrated brine solution. The aqueous layer was then extracted with CH_2Cl_2 (x2) and the combined organic phases were dried using Na_2SO_4, filtered and concentrated under reduced pressure. The product was used in the following step without further purification.[2] (**Note** – methyl, ethyl and isopropyl isoxazolones should be stored below zero or used immediately, or else decomposition occurs)

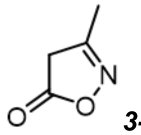

3-methylisoxazol-5(4H)-one was prepared according to procedure **A**. 1 g of ketoester was used and 813 mg of product was obtained as a pale yellow oil (95% yield). Spectral data matched reported literature data.[3] ^{1}H NMR (400 MHz, $CDCl_3$) δ 3.39 (s, 2H), 2.15 (s, 3H).

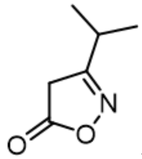

3-isopropylisoxazol-5(4H)-one was prepared according to procedure **A**. 2 g of ketoester was used and 1.70 g of product was obtained as a colorless oil (96% yield). Spectral data matched reported literature data.[3] ^{1}H NMR (400 MHz, $CDCl_3$) δ 3.38 (s, 2H), 2.78 (hept, J = 6.9 Hz, 1H), 1.23 (d, J = 6.9 Hz, 6H).

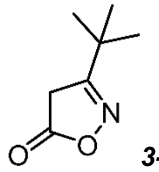

3-tert-butylisoxazol-5(4H)-one was prepared according to procedure **A**. 3 g of ketoester was used and 2.21 g of product was obtained as a white solid (90% yield). Spectral data matched reported literature data.[4] ^{1}H NMR (400 MHz, $CDCl_3$) δ 3.40 (s, 2H), 1.25 (s, 9H).

General Procedure B: Knoevenagel Condensation

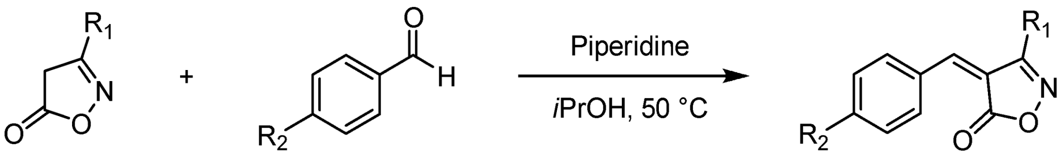

Isoxazolone (1 equiv.), aldehyde (1.2 equiv.) and *i*PrOH (0.5 M in respect to isoxazolone) were added to a round bottom with stirring, followed by piperidine (5 µL/mmol isoxazolone). The solution was heated to 50 °C and monitored by TLC until completion (*ca.* 3 – 5 hr). Products precipitated out as a bright to pale yellow solid and were isolated by filtration and washed with a cold 1:1 *i*PrOH/H_2O mixture.

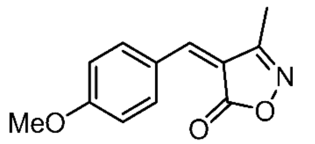

(Z)-4-(4-methoxybenzylidene)-3-methylisoxazol-5(4H)-one ($\boldsymbol{1_{Me,OMe}}$) was prepared according to general procedure **B**. 400 mg of isoxazolone was used and 152 mg of product was obtained as a yellow solid (17% yield). Spectral data matched reported literature data.[5] ^{1}H NMR (400 MHz, DMSO-*d*$_6$) δ 8.53 (d, 8.0 Hz, 2H), 7.88 (s, 1H), 7.17 (d, 8.0 Hz, 2H), 3.90 (s, 3H), 2.27 (s, 3H).

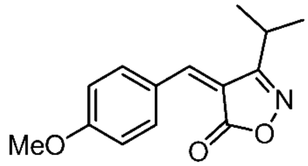

(Z)-4-(4-methoxybenzylidene)-3-isopropylisoxazol-5(4H)-one ($\boldsymbol{1_{iPr,OMe}}$) was prepared according to general procedure **B**. 500 mg of isoxazolone was used and 447 mg of product was obtained as a yellow solid (46% yield). Spectral data matched reported literature data.[6] ^{1}H NMR (400 MHz, DMSO-*d*$_6$) δ 8.55 (d, 12 Hz, 2H), 7.94 (s, 1H), 7.16 (d, 8.0 Hz, 2H), 3.90 (s, 3H), 3.22 (hept, *J* = 6.8 Hz, 1H), 1.27 (d, *J* = 6.9 Hz, 6H).

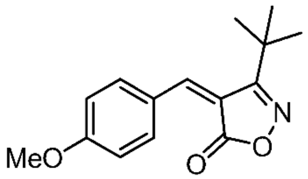

(Z)-4-(4-methoxybenzylidene)-3-tert-butylisoxazol-5(4H)-one ($\boldsymbol{1_{tBu,OMe}}$) was prepared according to general procedure **B**. 150 mg of isoxazolone was used and 48 mg of product was obtained as a yellow solid (17% yield). ^{1}H NMR (400 MHz, DMSO-*d*$_6$) δ 8.54 (d, 8.0 Hz, 2H), 8.10 (s, 1H), 7.13 (d, 8.0 Hz, 2H), 3.90 (s, 3H), 1.41 (s, 9H). ^{13}C NMR (101 MHz, DMSO-*d*$_6$) δ 169.42, 169.08, 164.04, 151.75, 137.09, 125.27, 114.32, 113.65, 55.83, 34.29, 28.24. HRMS (ESI) calcd for $C_{15}H_{17}NO_3$ $[M + H]^+$ *m/z* 260.1287, found 260.1274.

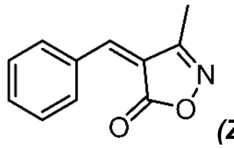

(Z)-4-benzylidene-3-methylisoxazol-5(4H)-one ($\boldsymbol{1_{Me,H}}$) was prepared according to general procedure **B**. 850 mg of isoxazolone was used and 536 mg of product was obtained as a pale yellow solid (33% yield). Spectral data matched reported literature data.[5] ^{1}H NMR (400 MHz, DMSO-*d*$_6$) δ 8.45 – 8.38 (m, 2H), 7.70 – 7.63 (m, 1H), 7.63 – 7.55 (m, 2H), 2.30 (s, 3H).

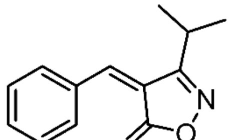

(Z)-4-benzylidene-3-isopropylisoxazol-5(4H)-one ($\boldsymbol{1_{iPr,H}}$) was prepared according to general procedure **B**. 882 mg of isoxazolone was used and 355 mg of product was obtained as a pale yellow solid (24% yield). Spectral data matched reported literature data.[6] ^{1}H NMR (400 MHz, DMSO-*d*$_6$) δ 8.45 – 8.37 (m, 2H), 8.04 (s, 1H), 7.68 – 7.62 (m, 1H), 7.58 (ddt, *J* = 8.3, 6.8, 1.3 Hz, 2H), 3.25 (hept, *J* = 6.9 Hz, 1H), 1.28 (d, *J* = 6.9 Hz, 6H).

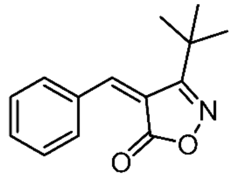

***(Z)*-4-benzylidene-3-*tert*-butylisoxazol-5(4H)-one (1$_{tBu,H}$)** was prepared according to general procedure **B**. 1 g of isoxazolone was used and 904 mg of product was obtained as a pale yellow solid (56% yield). Spectral data matched reported literature data.[7] ^{1}H NMR (400 MHz, DMSO-*d$_6$*) δ 8.35 – 8.28 (m, 2H), 8.22 (s, 1H), 7.66 – 7.59 (m, 1H), 7.59 – 7.51 (m, 2H), 1.42 (s, 9H).

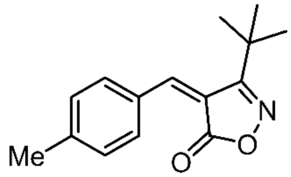

***(Z)*-4-(4-methylbenzylidene)-3-*tert*-butylisoxazol-5(4H)-one (1$_{tBu,Me}$)** was prepared according to general procedure **B**. 150 mg of isoxazolone was used and 190 mg of product was obtained as a yellow solid (74% yield). Spectral data matched reported literature data.[8] ^{1}H NMR (400 MHz, DMSO-*d$_6$*) δ 8.30 (d, 8.0 Hz, 2H), 8.15 (s, 1H), 7.37 (d, *J* = 8.0 Hz, 2H), 2.41 (s, 3H), 1.41 (s, 9H).

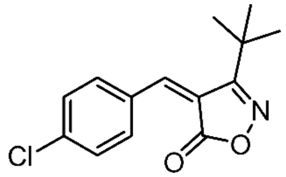

***(Z)*-4-(4-chlorobenzylidene)-3-*tert*-butylisoxazol-5(4H)-one (1$_{tBu,Cl}$)** was prepared according to general procedure **B**. 150 mg of isoxazolone was used and 132 mg of product was obtained as a pale yellow solid (47% yield). ^{1}H NMR (400 MHz, DMSO-*d$_6$*) δ 8.33 (d, 8.0 Hz, 2H), 8.21 (s, 1H), 7.63 (d, 8.0 Hz, 2H), 1.41 (s, 9H). ^{13}C NMR (101 MHz, DMSO-*d$_6$*) δ 169.17, 168.10, 150.54, 137.93, 134.99, 130.76, 128.51, 117.85, 34.39, 28.09. HRMS (ESI) calcd for $C_{14}H_{14}ClNO_2$ [M + H]$^+$ *m/z* 264.0791, found 260.0780.

Synthesis of Ditopic Michael Acceptors

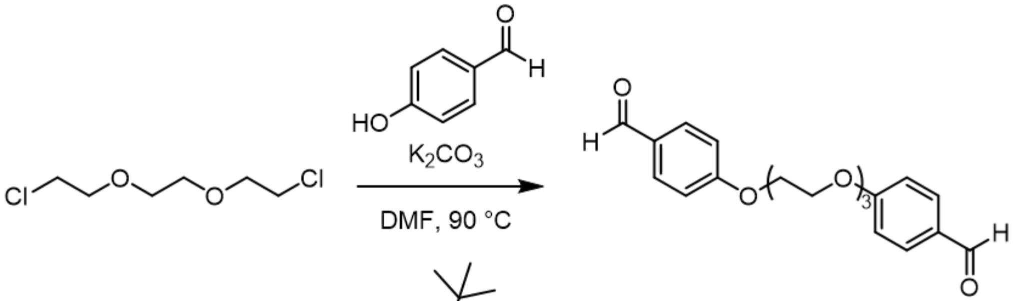

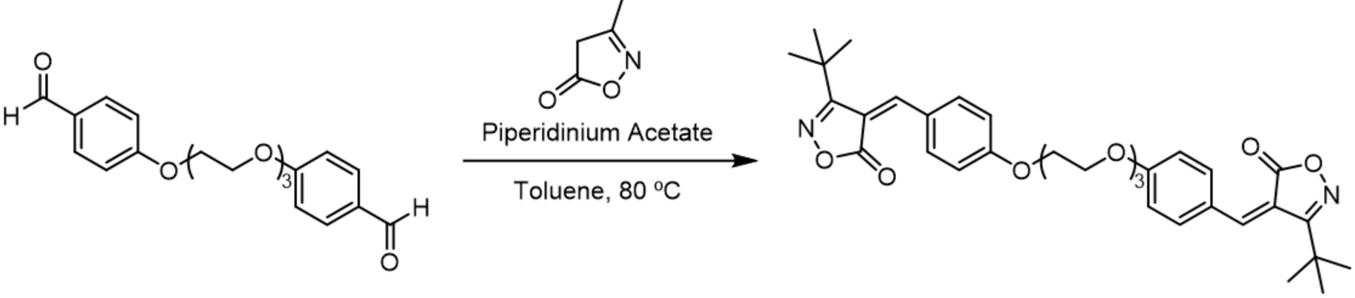

4,4'-(((ethane-1,2-diylbis(oxy))bis(ethane-2,1-diyl))bis(oxy))dibenzaldehyde

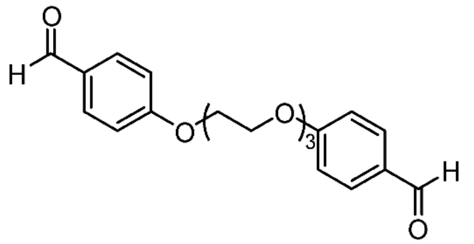

1,2-bis-(2-chloroethoxyl)ethane (1 equiv.), 4-hydroxybenzaldehyde (3 equiv.) and potassium carbonate (4 equiv.) were added to a round bottom charged with DMF (0.2 M in respect to 1,2-bis-92-chloroethoxyl(ethane)). The solution was heated to 90 °C and stirred overnight. After, the mixture was washed twice with water, followed by saturated aqueous $NaHCO_3$. The organic layer was then dried with $MgSO_4$, concentrated under reduced pressure, and purified by column chromatography (1:1 hexanes/EtOAc). 5 g of 1,2-bis-92-chloroethoxyl(ethane) was used and 8.23 g of product was obtained as a white powder (86% yield). Spectral data matched reported literature data.[9] ^{1}H NMR (500 MHz, $CDCl_3$) δ 9.88 (s, 2H), 7.85 – 7.79 (m, 4H), 7.04 – 6.98 (m, 4H), 4.23 – 4.18 (m, 4H), 3.92 – 3.87 (m, 4H), 3.76 (s, 4H).

(4Z,4'Z)-4,4'-(((((ethane-1,2-diylbis(oxy))bis(ethane-2,1-diyl))bis(oxy))bis(4,1-phenylene))bis(methaneylylidene))bis(3-(tert-butyl)isoxazol-5(4H)-one) (2$_{tBu}$)

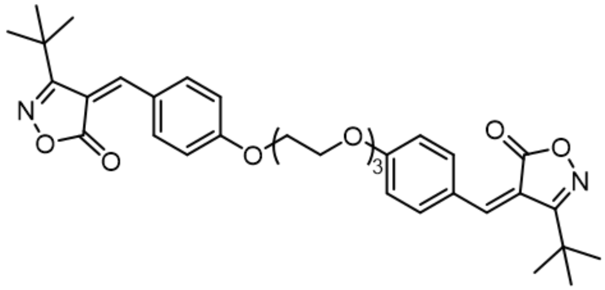

A round bottom was charged with dialdehyde (1 equiv.), isoxazolone (3 equiv.) and toluene (0.01 M in respect to dialdehyde) and allowed to stir. Then, a small scoop of piperidinium acetate (~0.1 equiv.) was added. The solution was heated to 80 °C and closely monitored by TLC until completion. The reaction mixture was then concentrated under reduced pressure and purified using silica gel column chromatography (4:1 hexanes/EtOAc → 100% EtOAc). 150 mg of dialdehyde was used and 230 mg of product was obtained as a yellow powder (91% yield). ^{1}H NMR (500 MHz, $CDCl_3$) δ 8.38 – 8.31 (m, 4H), 7.76 (s, 2H), 7.03 – 6.96 (m, 4H), 4.26 – 4.20 (m, 4H), 3.93 – 3.87 (m, 4H), 3.76 (s, 4H), 1.45 (s, 18H). ^{13}C NMR (101 MHz, $CDCl_3$) δ 169.61, 169.37, 163.60, 150.70, 136.95, 125.75, 115.25, 115.11, 71.12, 69.66, 67.97, 34.78, 28.90. HRMS (ESI) calcd for $C_{34}H_{41}N_2O_8$ $[M + H]^+$ *m/z* 605.2863, found 605.2866.

1D. Gel Formation

To prepare gel samples, 625 mg of 4-armed PEG-SH (10 kDa) was dissolved in ~4.3 mL of DMSO (targeting 50 mM thiol end groups, accounting for the density of PEG). After dissolving completely, approximately 76 mg of **2OMe$_{tBu}$** was added to the solution targeting 50 mM of Michael acceptor end groups. After thorough sonication and mixing, the sample vial was covered with aluminum foil and was equilibrated for at least 24 hours (dark, room temperature) prior to measurement.

2. CHARACTERIZATION

2A. UV-Vis

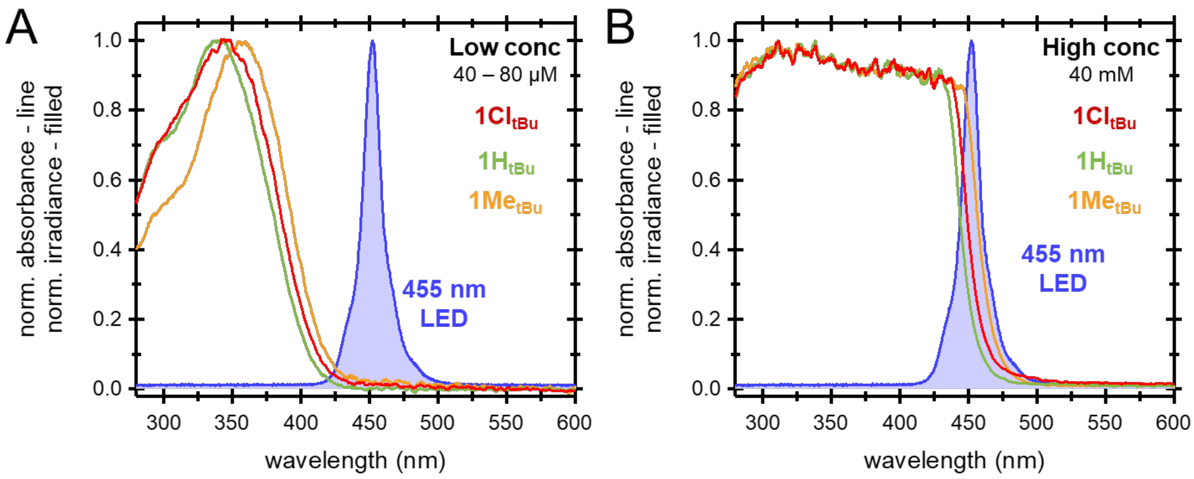

Figure S1: Absorbance spectra of **1Cl$_{tBu}$** (red), **1H$_{tBu}$** (green), and **1Me$_{tBu}$** (orange) at (A) 40 – 80 µM and (B) 40 mM overlayed with 455 nm LED irradiance spectrum.

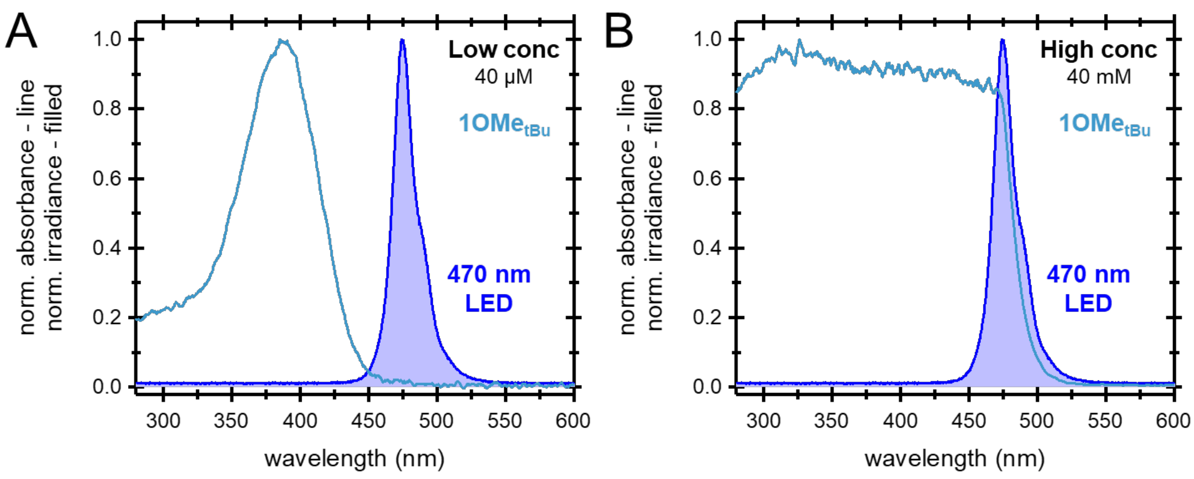

Figure S2: Absorbance spectra of **1OMe$_{tBu}$** (blue) at (A) 40 – 80 µM and (B) 40 mM overlayed with 470 nm LED irradiance spectrum.

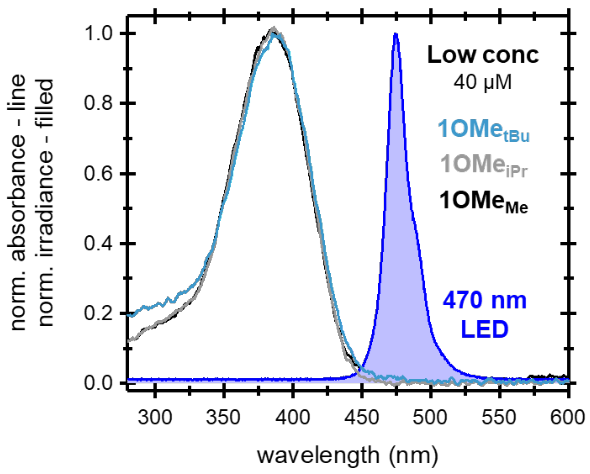

Figure S3: Absorbance spectra of **1OMe*tBu*** (blue), **1OMe*iPr*** (grey), and **1OMeMe** (black) at 40 µM and overlayed with 470 nm LED irradiance spectrum.

2B. NMR Analysis

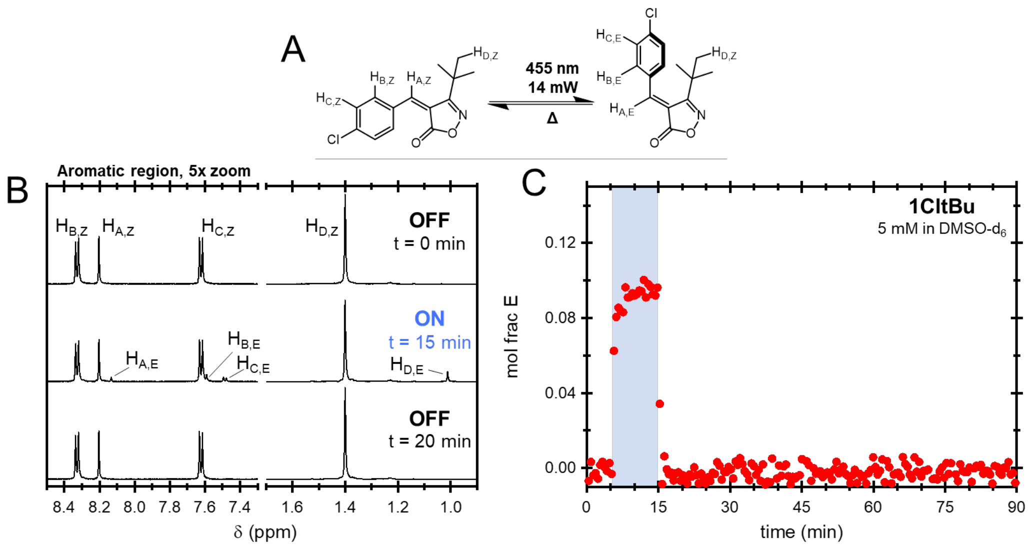

Figure S4: (A) Equilibrium between isomers of **1Cl$_{tBu}$** during irradiation with 455 nm light. (B) Relevant regions of ^{1}H NMR spectrum of **1Cl$_{tBu}$** at select time points during irradiation demonstrating the rapid generation and loss of *E*-isomer peaks during and after exposure to 455 nm light. (C) Mole fraction of *E* isomer before, during, and after irradiation. Even without thiol, the generated *E* isomer rapidly reverts to the stable *Z* isomer after irradiation.

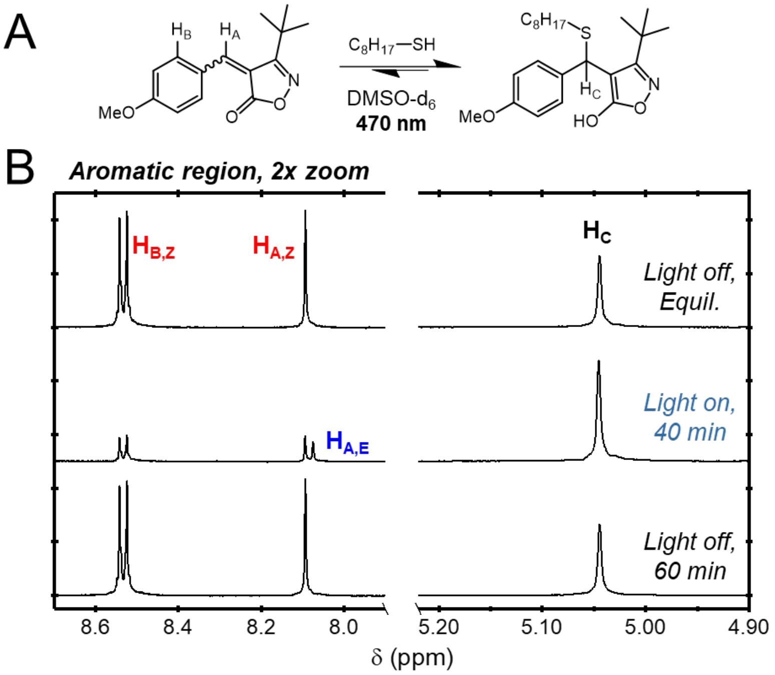

Figure S5: (A) Equilibrium of **1OMe$_{tBu}$** and 1-octanethiol. (B) Relevant regions of ^{1}H NMR spectrum of **1OMe$_{tBu}$** equilibrated with 1-octanethiol demonstrating decreases of peaks $H_{B,Z}$ and $H_{A,Z}$ and increase in peaks $H_{A,E}$ and H_C during irradiation with 455 nm light and reversion after ceasing exposure to light.

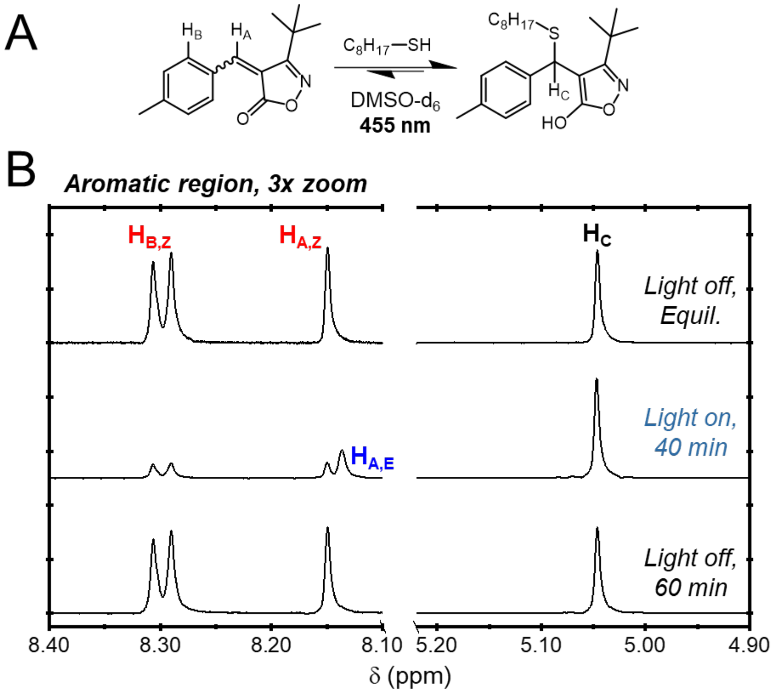

Figure S6: (A) Equilibrium of **1Me$_{tBu}$** and 1-octanethiol. (B) Relevant regions of ^{1}H NMR spectrum of **1OMe$_{tBu}$** equilibrated with 1-octanethiol demonstrating decreases of peaks $H_{B,Z}$ and $H_{A,Z}$ and increase in peaks $H_{A,E}$ and H_C during irradiation with 455 nm light and reversion after ceasing exposure to light.

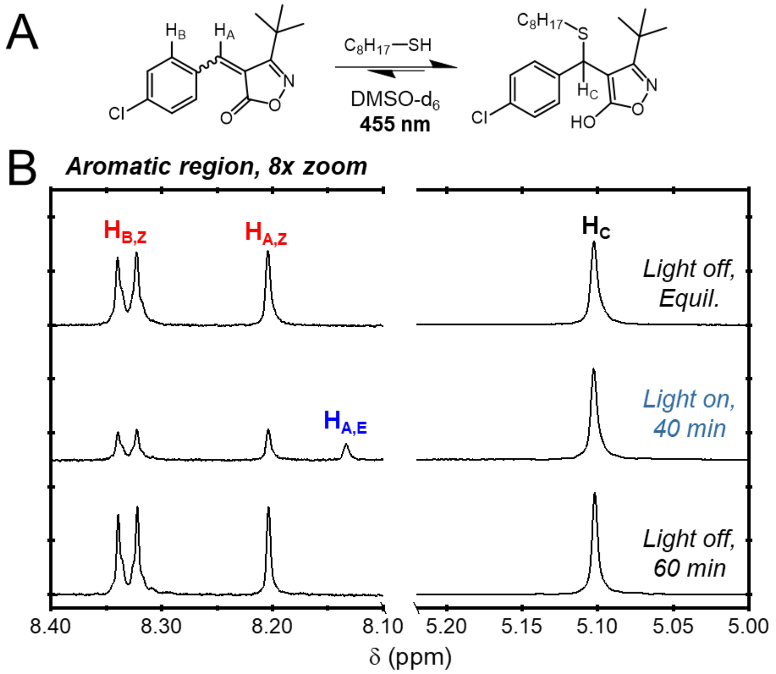

Figure S7: (A) Equilibrium of **1Cl$_{tBu}$** and 1-octanethiol. (B) Relevant regions of ^{1}H NMR spectrum of **1OMe$_{tBu}$** equilibrated with 1-octanethiol demonstrating decreases of peaks $H_{B,Z}$ and $H_{A,Z}$ and increase in peaks $H_{A,E}$ and H_C during irradiation with 455 nm light and reversion after ceasing exposure to light.

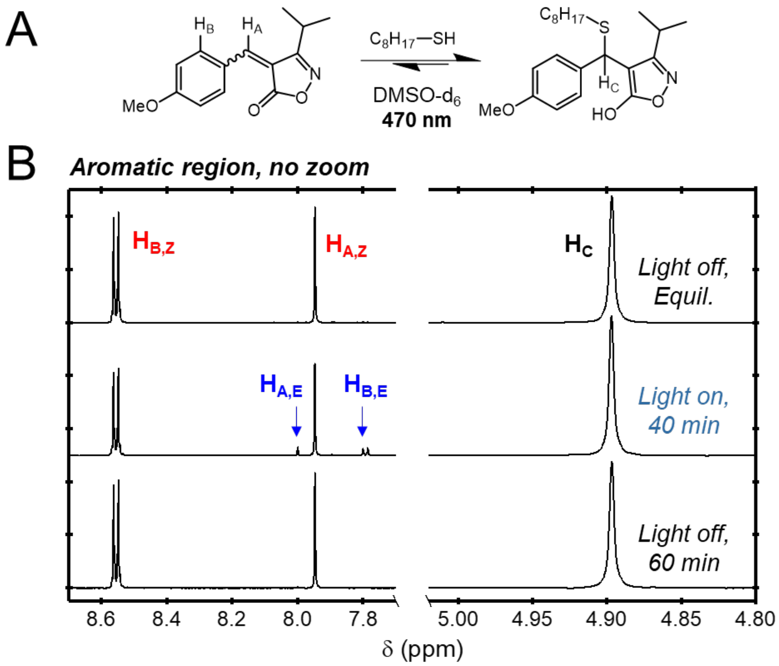

Figure S8: (A) Equilibrium of **1OMe*iPr*** and 1-octanethiol. (B) Relevant regions of ^{1}H NMR spectrum of **1OMe*tBu*** equilibrated with 1-octanethiol demonstrating decreases of peaks $H_{B,Z}$ and $H_{A,Z}$ and increase in peaks $H_{A,E}$ and H_C during irradiation with 455 nm light and reversion after ceasing exposure to light.

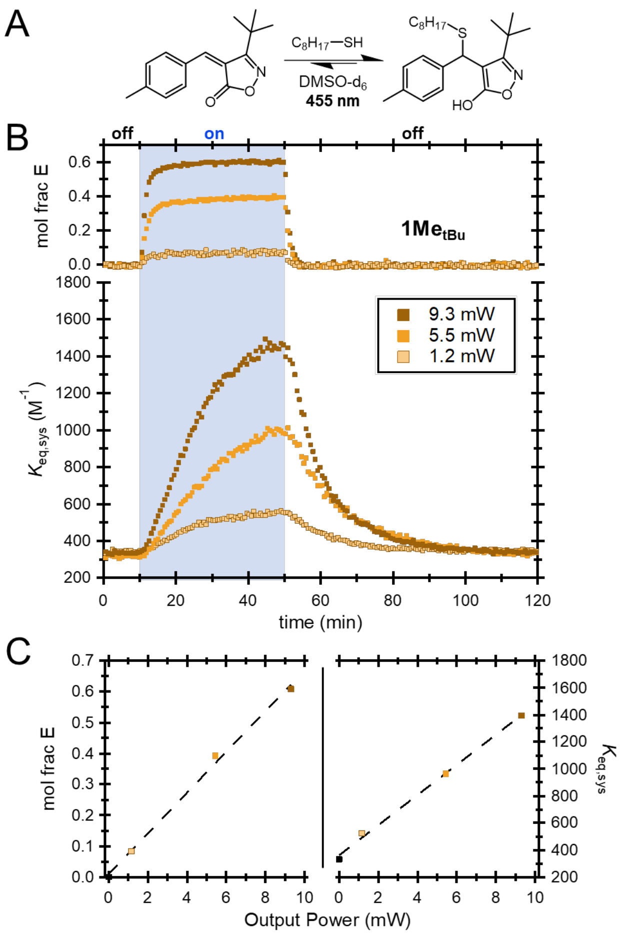

Figure S9: (A) Equilibrium between **1Me$_{tBu}$** and 1-octanethiol. (B) Mole fraction of *E* isomer and overall system K_{eq} of **1Me$_{tBu}$** equilibrated with 1-octanethiol during NMR photoisomerization experiment at varying intensities of light (1.2 to 9.3 mW/cm^2). (C) Mole fraction of *Z* isomer and overall system K_{eq} versus light intensity.

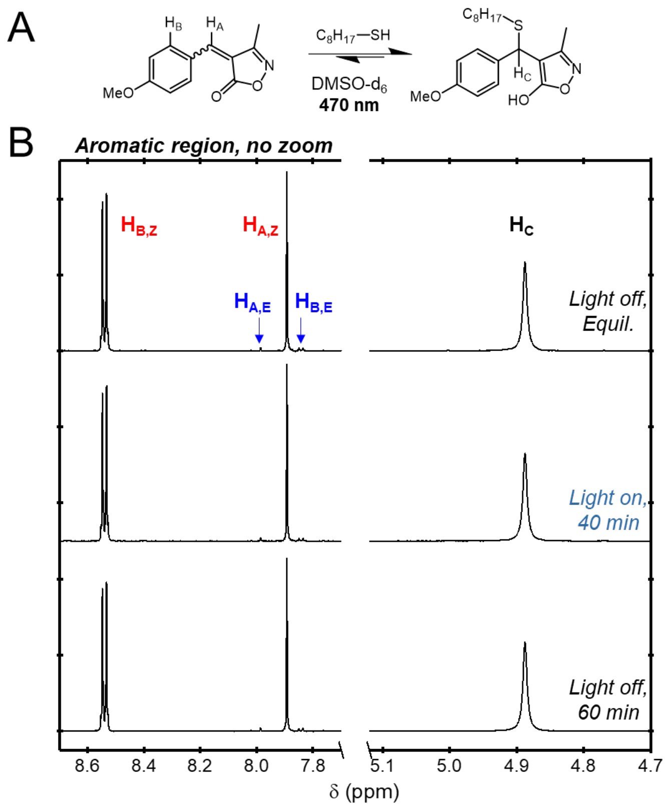

Figure S10: (A) Equilibrium of **1OMe$_{Me}$** and 1-octanethiol. (B) Relevant regions of ^{1}H NMR spectrum of **1OMe$_{tBu}$** equilibrated with 1-octanethiol demonstrating decreases of peaks $H_{B,Z}$ and $H_{A,Z}$ and increase in peaks $H_{A,E}$ and H_C during irradiation with 455 nm light and reversion after ceasing exposure to light.

Table S1: Extent of isomerization across species and measured irradiation conditions, all samples measured in 50 mM conditions with a stoichiometric amount of octanethiol in DMSO-d$_6$.

Species	Wavelength (nm)	Power (mW)	Max mol frac E isomer	Max $K_{eq,sys}$ (M^{-1})
**1OMe$_{tBu}$**	470	37.6	0.449	801
**1OMe$_{tBu}$**	470	14	0.165	430
**1OMe$_{tBu}$**	470	3.2	0.043	147
**1Me$_{tBu}$**	455	9.3	0.600	1471
**1Me$_{tBu}$**	455	5.5	0.393	1012
**1Me$_{tBu}$**	455	1.2	0.071	564
**1H$_{tBu}$**	455	14	0.357	3460
**1Cl$_{tBu}$**	455	14	0.366	7440
**1OMe$_{iPr}$**	470	14	0.111	118
**1OMe$_{Me}$**	470	14	0.021	88

2C. Computational Details

Density functional theory (DFT) calculations were performed using the Gaussian 16 package with the M06-2X/6-311+G(d,p) level of theory.[10] The solvent environment was simulated using the PCM, CPCM, or SMD continuum solvent model.[11–14] The stable conformations of the molecules were found by energy minimization. Gibbs free energies are calculated based on harmonic vibration frequencies, reported at a standard state of 1 mol/L and 25 °C. As these room temperature dynamic reactions have relatively small reaction energies (ΔG_{rxn}), the accuracy of the calculations were benchmarked against experimental data in our previous work.[15]

The optimized molecular configuration (xyz files) can be found in the ComputationalDataSet.zip file.

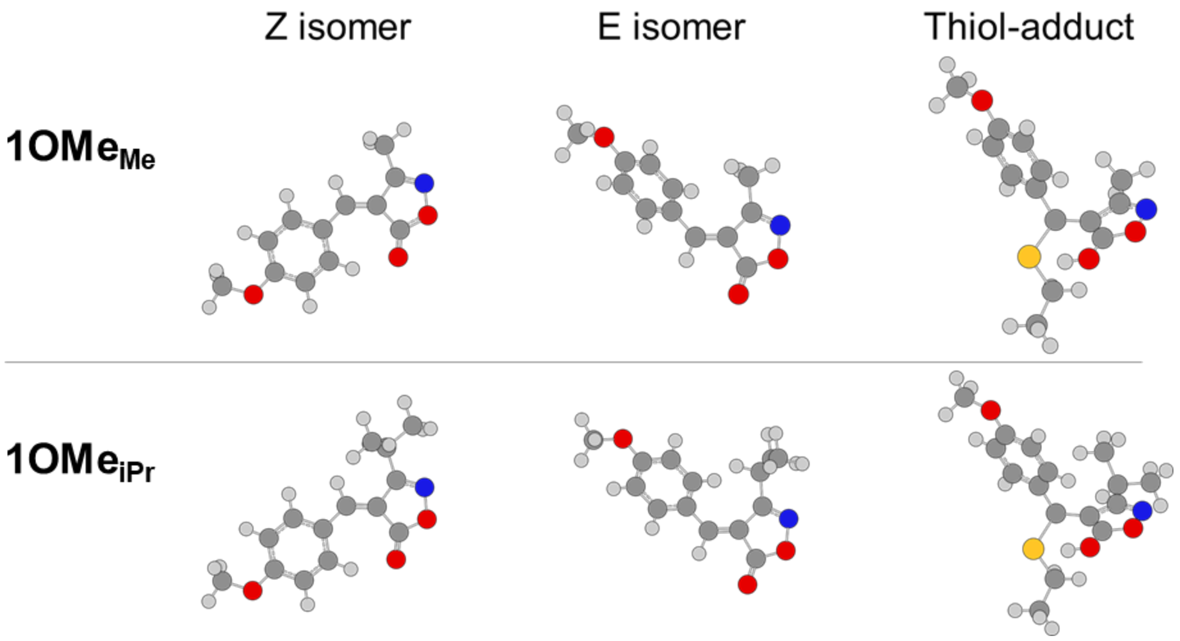

Figure S11: Conformations of **1OMe$_{Me}$** and **1OMe$_{iPr}$** and their respective thiol-adducts.

Table S2: Reaction Energies from DFT Calculations. Units for ΔH and ΔG are kJ/mol. ΔS is kJ/(mol· K)

	**Vacuum**			**CPCM**			**PCM**			**SMD**		
	**ΔH**	**ΔG**	**ΔS**	**ΔH**	**ΔG**	**ΔS**	**ΔH**	**ΔG**	**ΔS**	**ΔH**	**ΔG**	**ΔS**
E-1OMe$_{Me}$	-68.27	-20.20	-0.161	-54.48	-6.41	-0.161	-54.42	-6.31	-0.161	-51.47	-1.74	-0.167
Z-1OMe$_{Me}$	-60.04	-11.72	-0.162	-46.32	3.27	-0.166	-46.36	3.29	-0.167	-44.86	5.75	-0.170
E-1OMe$_{iPr}$	-74.67	-22.96	-0.173	-61.15	-12.03	-0.165	-61.12	-11.88	-0.165	-58.38	-8.38	-0.168
Z-1OMe$_{iPr}$	-62.94	-12.02	-0.171	-49.34	0.22	-0.166	-49.42	0.21	-0.166	-47.56	3.82	-0.17
E-1OMe$_{tBu}$	-86.22	-30.32	-0.187	-75.46	-18.71	-0.190	-75.40	-18.39	-0.191	-73.85	-16.57	-0.192
Z-1OMe$_{tBu}$	-65.31	-11.15	-0.182	-52.06	4.26	-0.189	-52.13	4.30	-0.189	-50.50	5.42	-0.188

Table S3: $\Delta\Delta G_{ZE}$ (in kJ/mol.) from DFT Calculations for Different Solvent Models.

$\Delta\Delta G_{ZE}$	Vacuum	CPCM	PCM	SMD	Average	Error
1OMe$_{Me}$	8.48	9.68	9.61	7.49	8.81	0.90
1OMe$_{iPr}$	10.94	12.25	12.09	12.20	11.87	0.54
1OMe$_{tBu}$	19.16	22.97	22.69	21.99	21.70	1.51

2D. Free Energy Calculations

Direct determination of $\Delta\Delta G_{Z,E}$ from residual *E*-isomers (**1OMe$_{iPr}$** and **1OMe$_{Me}$**)

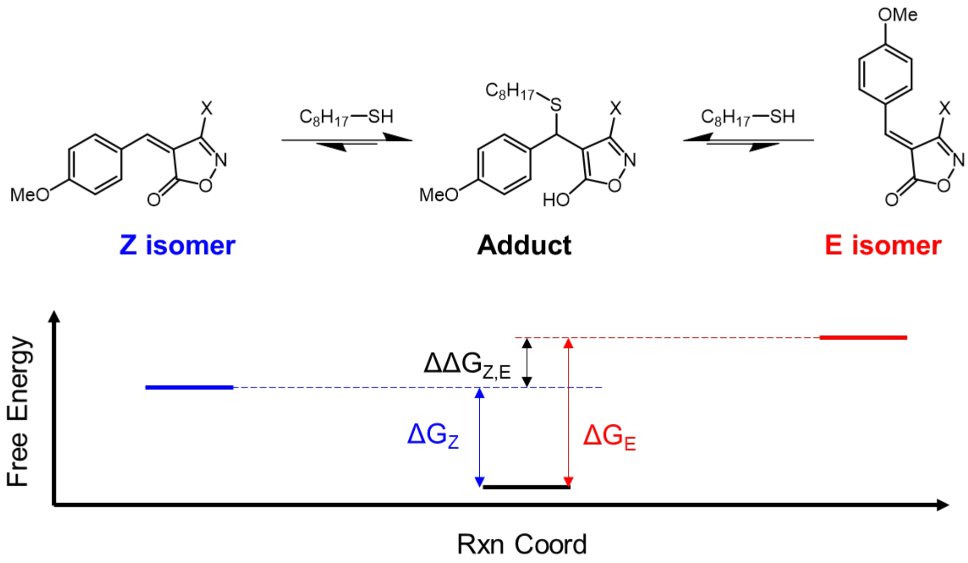

Scheme S1: Simplified energetic landscape between Z- and E-isomers of **1OMe$_X$** during dynamic exchange with octanethiol.

Consider the following system of equilibrium reactions:

R_1: Z-isomer + Thiol ⇌ Adduct

R_2: Adduct ⇌ E-isomer + Thiol

R_{net}: Z-isomer ⇌ E-isomer

A net equilibrium constant K can be defined as:

Eq S1: K = [E-isomer]/[Z-isomer]

The change in Gibbs free energy (in this case, the $\Delta\Delta G_{Z,E}$) is classically defined as:

Eq S2: $\Delta\Delta G_{Z,E} = -RT\ln(K)$

As an example, an equilibrated 40 mM mixture of 1OMe$_{Me}$ and octanethiol was found to have a consistent 0.021 mol fraction of E-isomer. This gives a K of ~0.021 and a corresponding $\Delta\Delta G_{Z,E}$ of ~9.6 kJ/mol.

Estimation of $\Delta\Delta G_{Z,E}$ from low intensity measurements ($1OMe_{tBu}$)

For sufficiently large values of $\Delta\Delta G_{Z,E}$, no residual E-isomer peaks can be resolved spectroscopically. As such, a low intensity of light was used to generate a small population of E isomer species (in an attempt to keep the system closer to equilibrium). The increase in system equilibrium constant ($K_{eq,sys}$) can then be used to calculate $\Delta\Delta G_{sys}$ via

Eq S3: $$\Delta\Delta G_{sys} = -RT\ln(K_Z) + RT\ln(K_{sys})$$

To estimate $\Delta\Delta G_{Z,E}$ the calculated value of $\Delta\Delta G_{sys}$ can then be normalized by the mol fraction of E-isomers (f_E):

Eq S4: $$\Delta\Delta G_{Z,E} \approx \Delta\Delta G_{sys}/f_E$$

As an example, for the low intensity data shown in main text Figure 4, the equilibrium K_Z value of ~85 M^{-1} was raised to ~136 M^{-1} after generating a mol fraction of E-isomers of ~0.045. As such, the calculated $\Delta\Delta G_{sys}$ was ~1164 kJ/mol and the estimated $\Delta\Delta G_{Z,E}$ was 25.9 kJ/mol.

2E. Shear Rheology

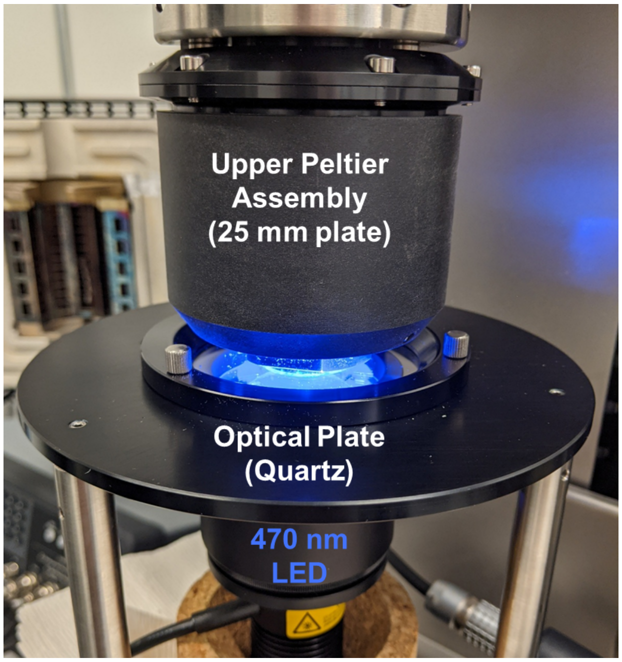

Figure S12: Shear rheology set-up for organogel photo-rheology experiment.

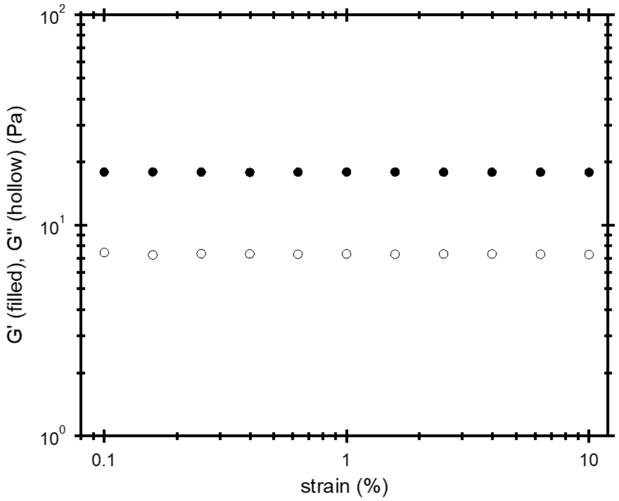

Figure S13: Amplitude sweep carried out before exposure to 470 nm light.

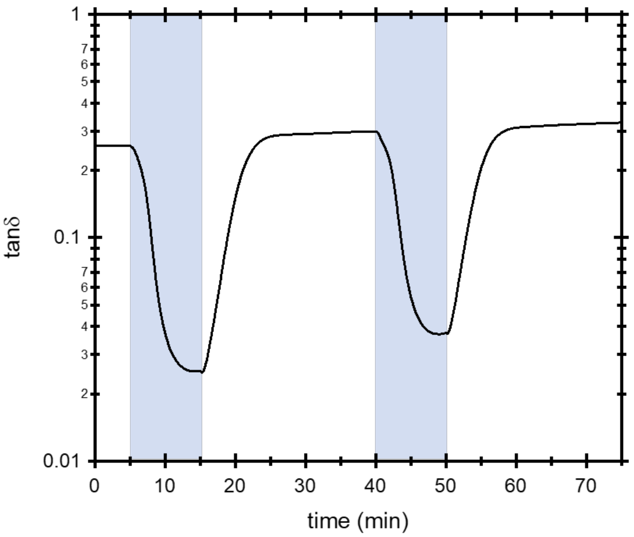

Figure S14: Measured tanδ of organogel during cycles of irradiation (470 nm) and darkness.

REFERENCES

1 N. D. Dolinski, Z. A. Page, F. Eisenreich, J. Niu, S. Hecht, J. Read de Alaniz and C. J. Hawker, *ChemPhotoChem*, 2017, **1**, 125–131.

2 A. E. Crolais, N. D. Dolinski, N. R. Boynton, J. M. Radhakrishnan, S. A. Snyder and S. J. Rowan, *J. Am. Chem. Soc.*, 2023, **145**, 14427–14434.

3 I. D. Jurberg and H. M. L. Davies, *Org. Lett.*, 2017, **19**, 5158–5161.

4 A. J. Kay, A. D. Woolhouse, G. J. Gainsford, T. G. Haskell, C. P. Wyss, S. M. Giffin, I. T. McKinnie and T. H. Barnes, *J. Mater. Chem.*, 2001, **11**, 2271–2281.

5 A. P. Chavan, A. B. Pinjari and P. C. Mhaske, *J. Heterocycl. Chem.*, 2015, **52**, 1911–1915.

6 K. Ablajan and H. Xiamuxi, *Synth. Commun.*, 2012, **42**, 1128–1136.

7 I. Nakamura, M. Okamoto and M. Terada, *Org. Lett.*, 2010, **12**, 2453–2455.

8 E. E. Galenko, M. S. Novikov and A. F. Khlebnikov, *J. Org. Chem.*, 2023, **88**, 8854–8864.

9 S. Nagarajan, M. Jeganathan Shanmugam and T. Mohan Das, *Carbohydr. Res.*, 2011, **346**, 722–727.

10 Y. Zhao and D. G. Truhlar, *Theor. Chem. Acc.*, 2008, **120**, 215–241.

11 M. Cossi, N. Rega, G. Scalmani and V. Barone, *J. Comput. Chem.*, 2003, **24**, 669–681.

12 Y. Takano and K. N. Houk, *J. Chem. Theory Comput.*, 2005, **1**, 70–77.

13 J. Tomasi, B. Mennucci and R. Cammi, *Chem. Rev.*, 2005, **105**, 2999–3094.

14 A. V. Marenich, C. J. Cramer and D. G. Truhlar, *J. Phys. Chem. B*, 2009, **113**, 6378–6396.

15 A. E. Crolais, C. Chen, J. Gao, N. D. Dolinski, Y. Xu, J. J. De Pablo, S. A. Snyder and S. J. Rowan, *J. Org. Chem.*, 2025, **90**, 4037–4045.

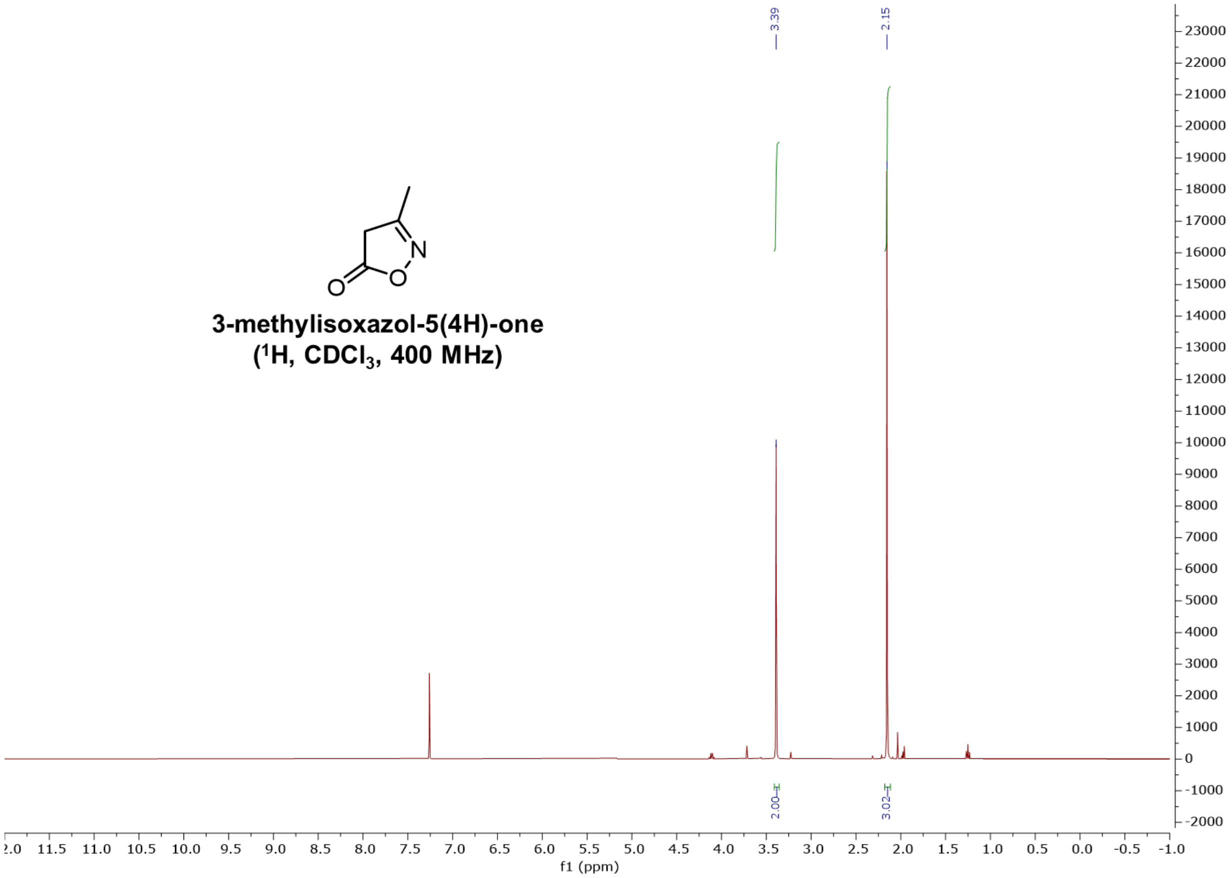

3-methylisoxazol-5(4H)-one
(^{1}H, $CDCl_3$, 400 MHz)

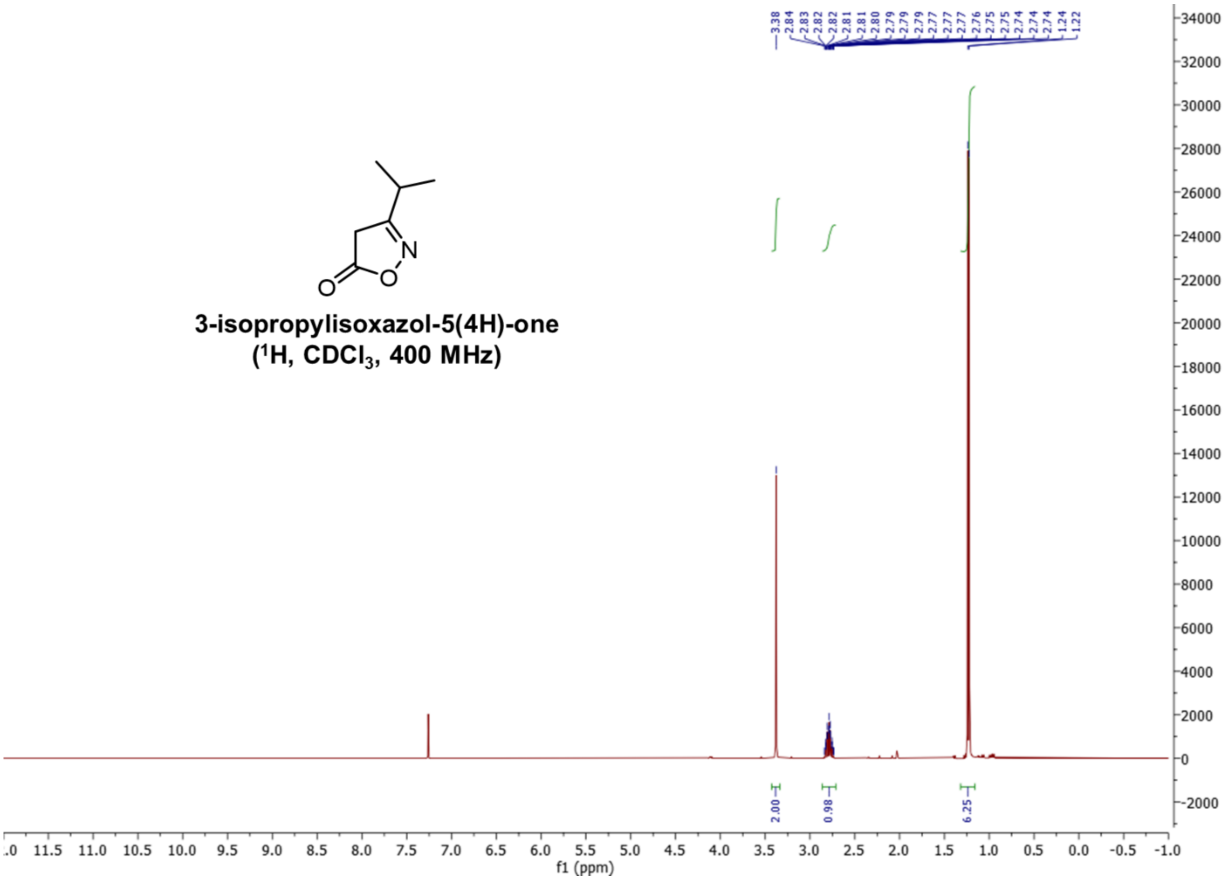

3-isopropylisoxazol-5(4H)-one
(^{1}H, $CDCl_3$, 400 MHz)

3-tert-butylisoxazol-5(4H)-one
(^{1}H, $CDCl_3$, 400 MHz)

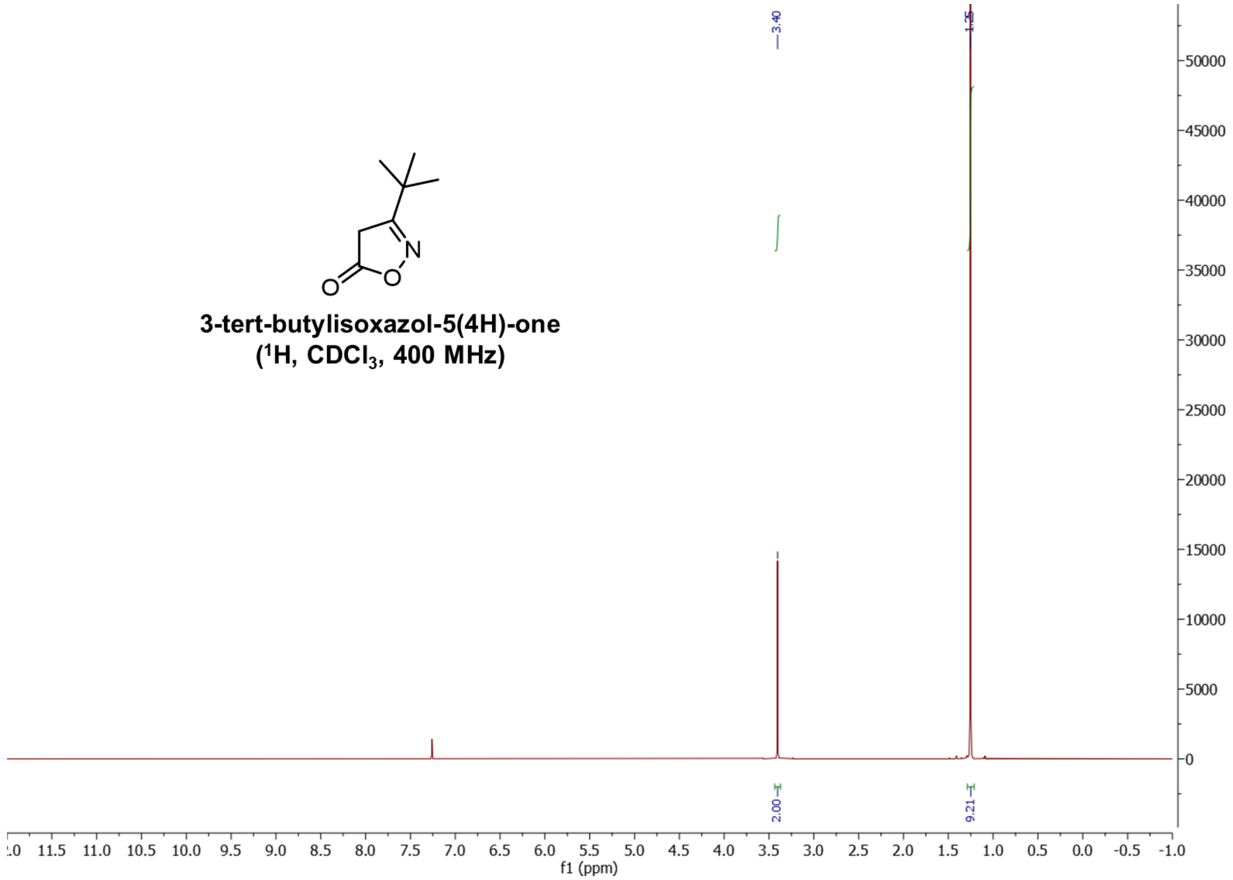

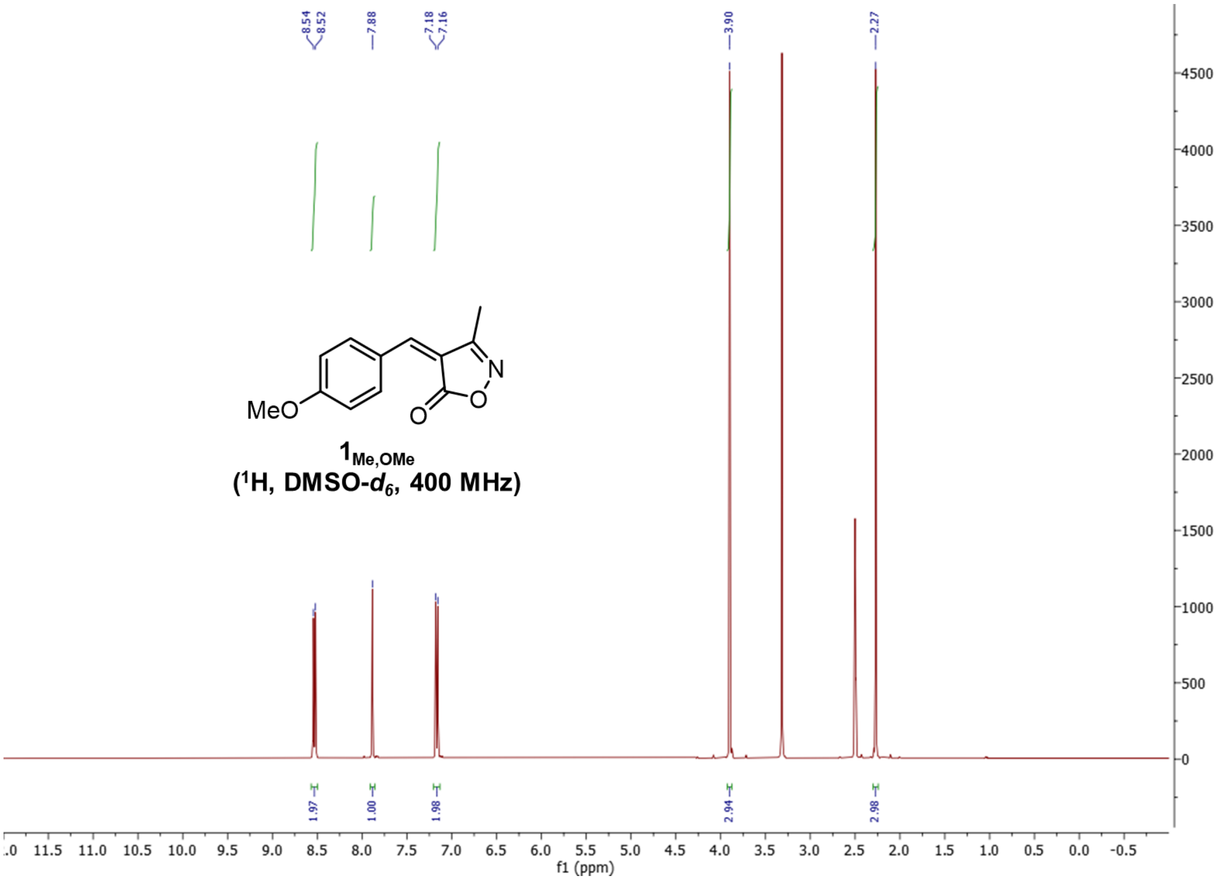

8.54
8.52
7.88
7.18
7.16
3.90
2.27
MeO
N
O
O
$1_{Me,OMe}$
(^{1}H, DMSO-d_6, 400 MHz)
1.97
1.00
1.98
2.94
2.98
4500
4000
3500
3000
2500
2000
1500
1000
500
0
11.5 11.0 10.5 10.0 9.5 9.0 8.5 8.0 7.5 7.0 6.5 6.0 5.5 5.0 4.5 4.0 3.5 3.0 2.5 2.0 1.5 1.0 0.5 0.0 -0.5
f1 (ppm)

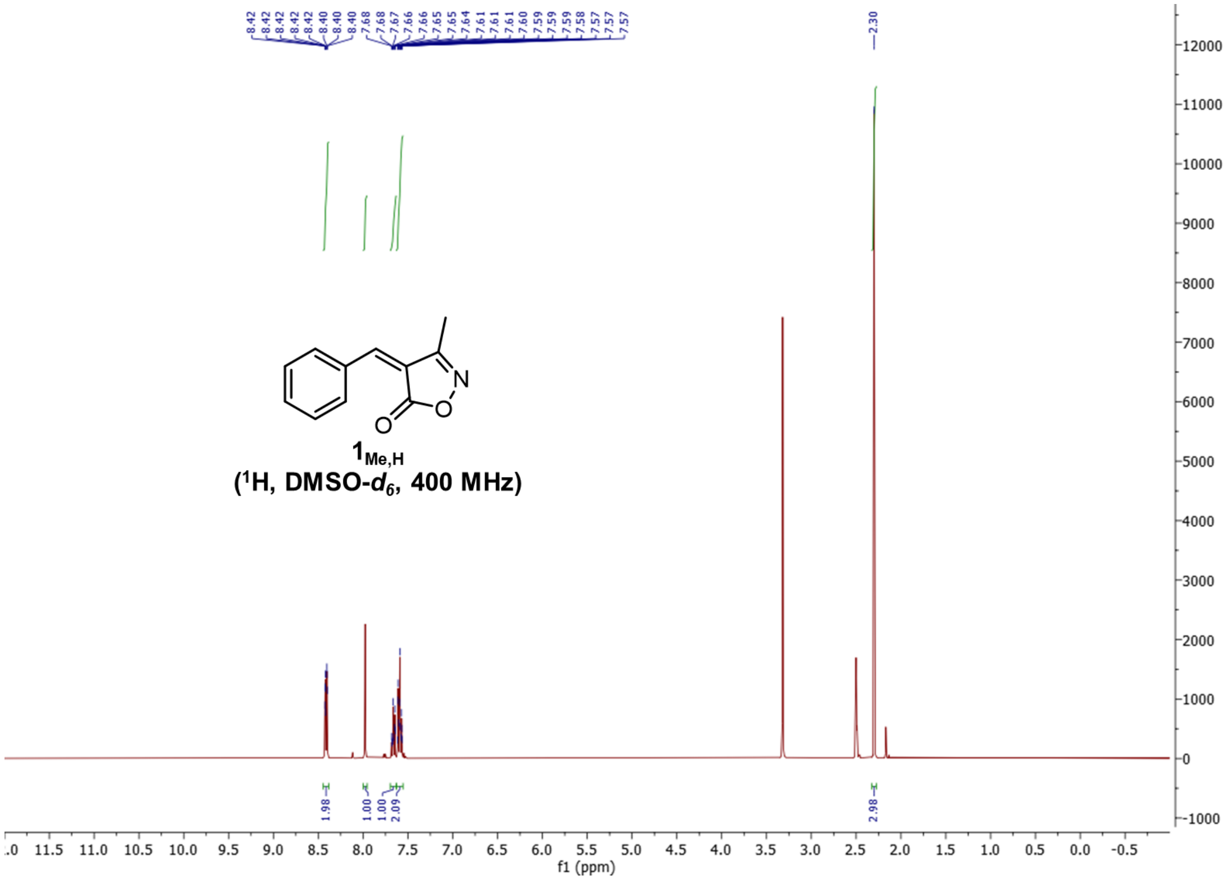
1Me,H
(1H, DMSO-d6, 400 MHz)
f1 (ppm)

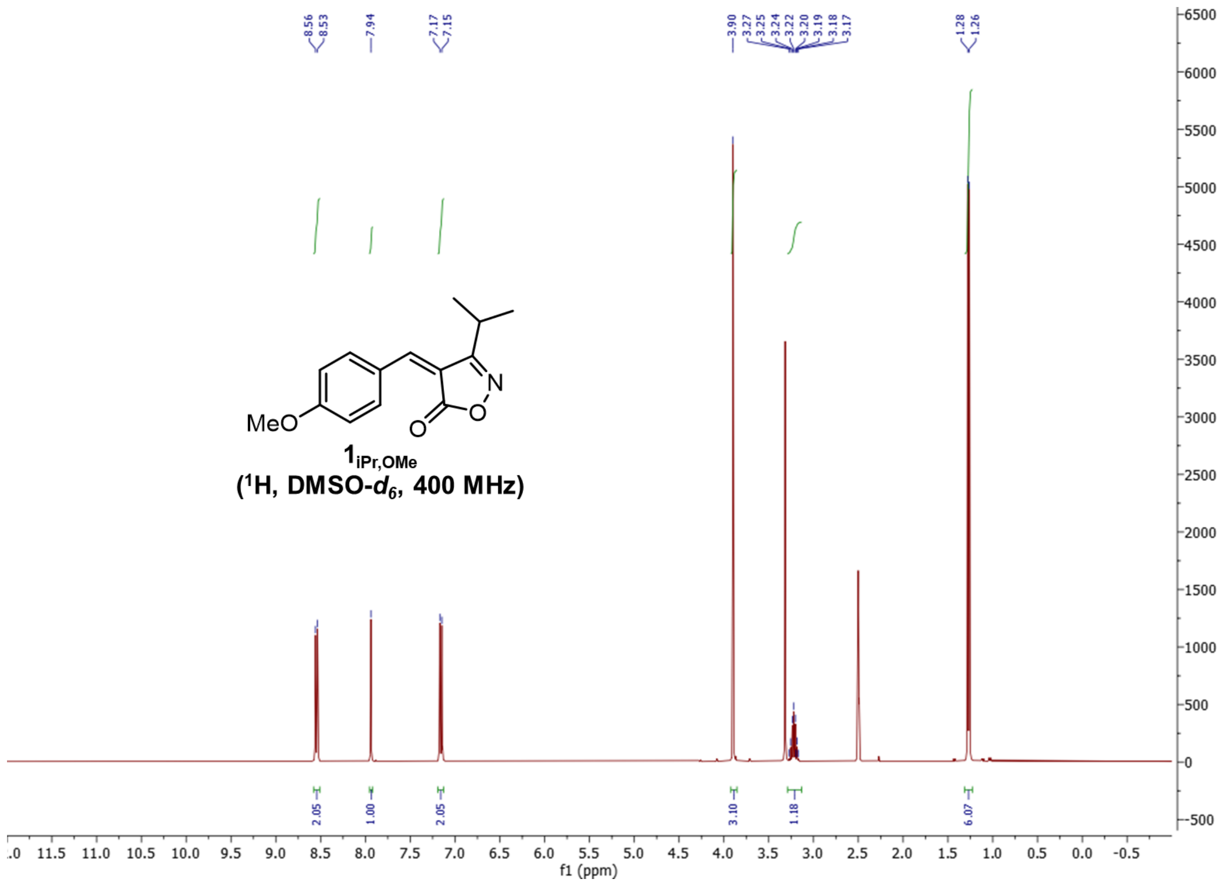

$\mathbf{1}_{iPr,OMe}$
(^{1}H, DMSO-d_6, 400 MHz)

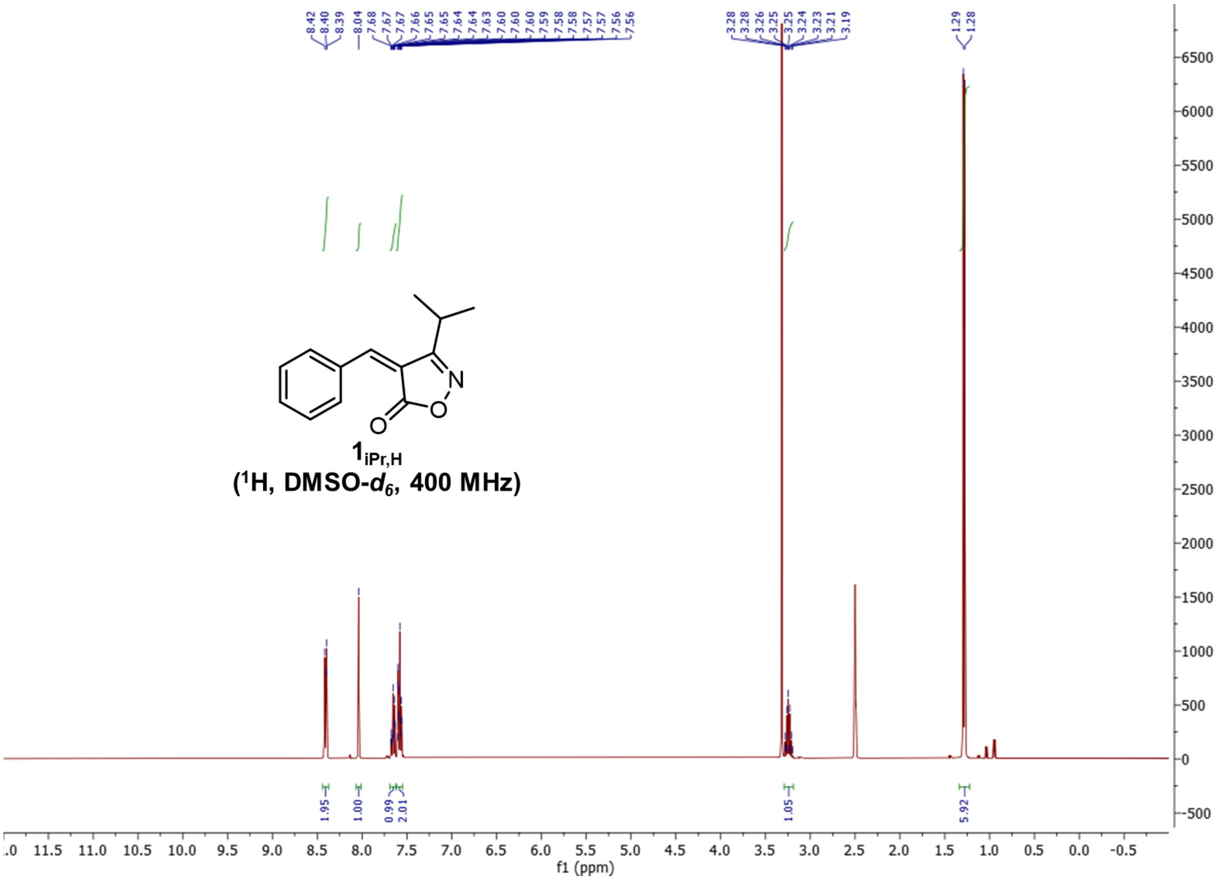
$\mathbf{1}_{iPr,H}$
(^{1}H, DMSO-d_6, 400 MHz)
f1 (ppm)

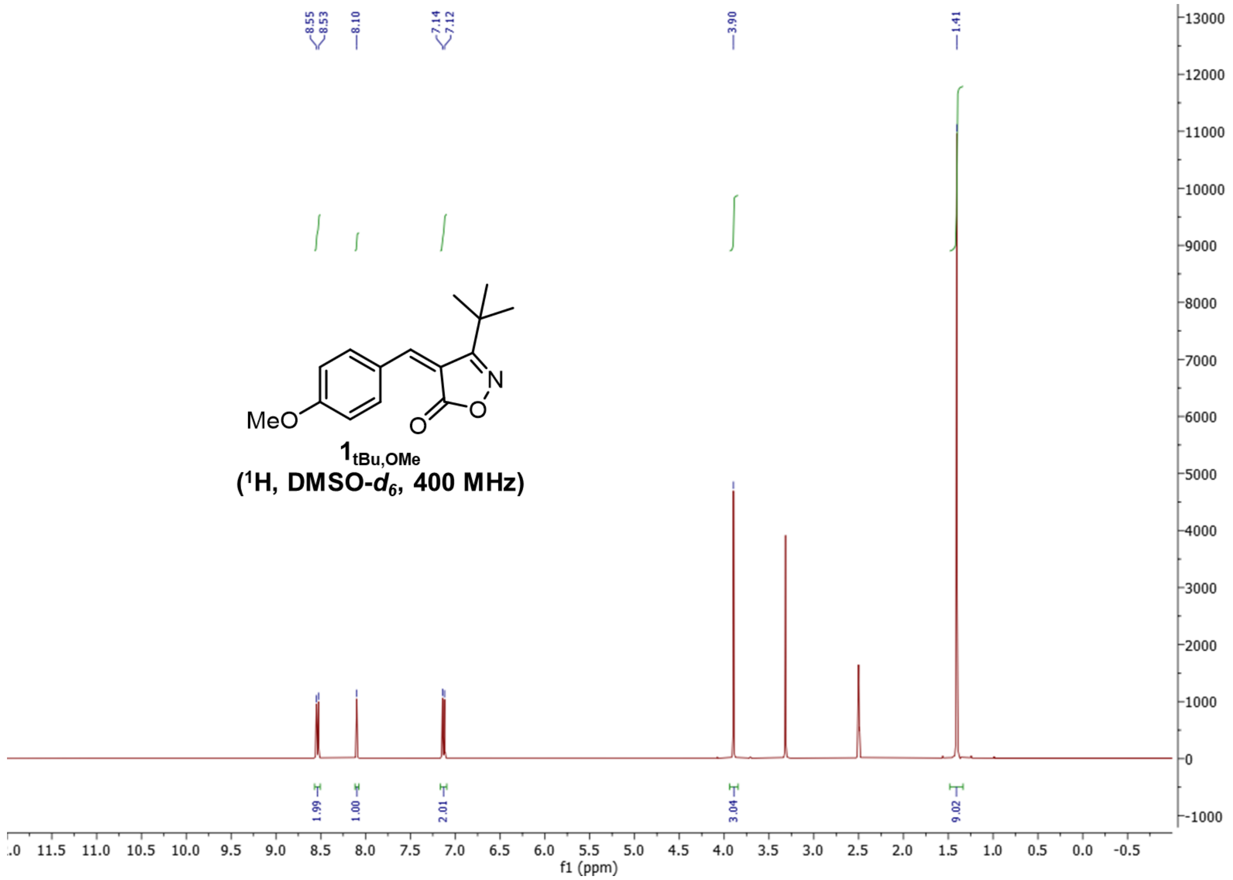

8.55
8.53
8.10
7.14
7.12
3.90
1.41
MeO
N
O
O
1tBu,OMe
(1H, DMSO-d6, 400 MHz)
1.99
1.00
2.01
3.04
9.02
f1 (ppm)

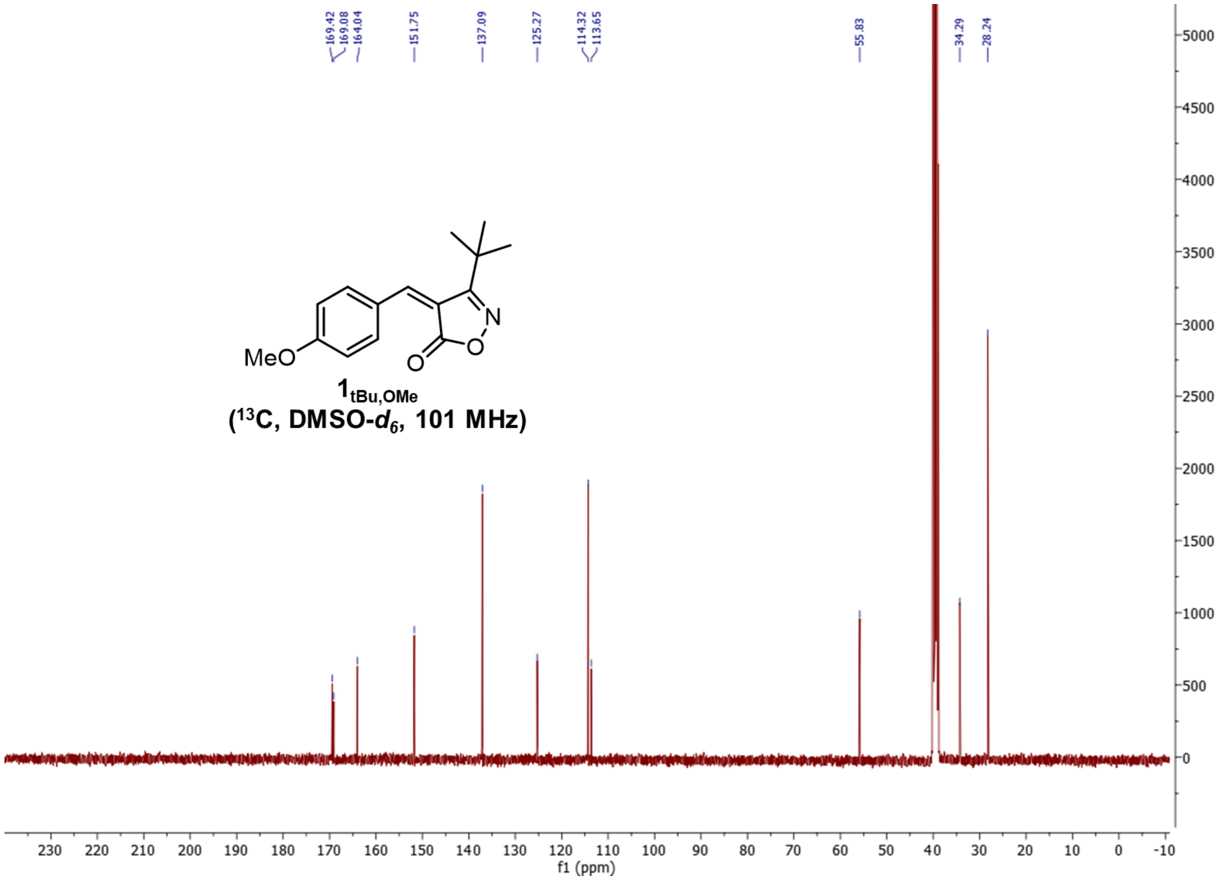
169.42
169.08
164.04
151.75
137.09
125.27
114.32
113.65
55.83
34.29
28.24
MeO
N
O
O
$1_{tBu,OMe}$
(^{13}C, DMSO-d_6, 101 MHz)
f1 (ppm)

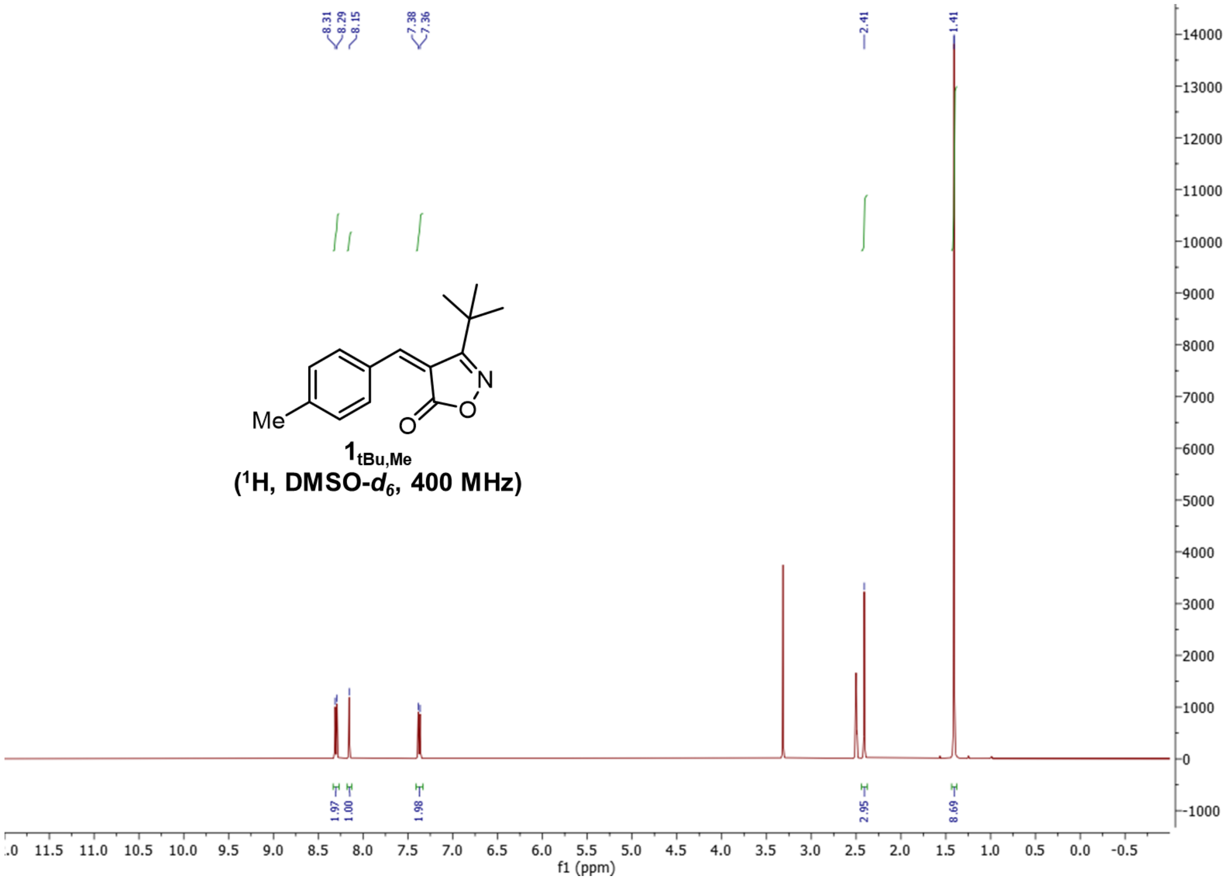
8.31
8.29
8.15
7.38
7.36
2.41
1.41
Me
N
O
O
$1_{tBu,Me}$
(^1H, DMSO-d_6, 400 MHz)
1.97
1.00
1.98
2.95
8.69
f1 (ppm)

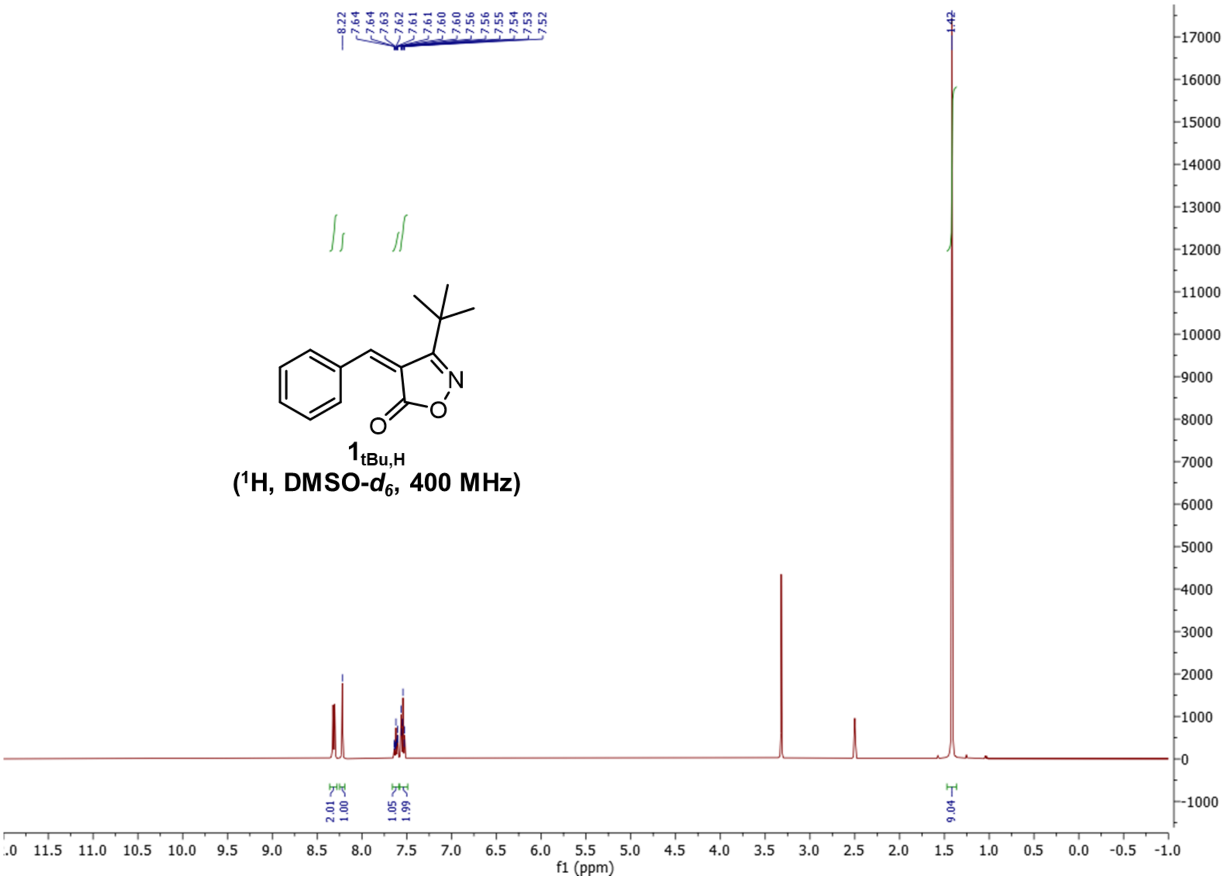

$\mathbf{1}_{tBu,H}$

(¹H, DMSO-d_6, 400 MHz)

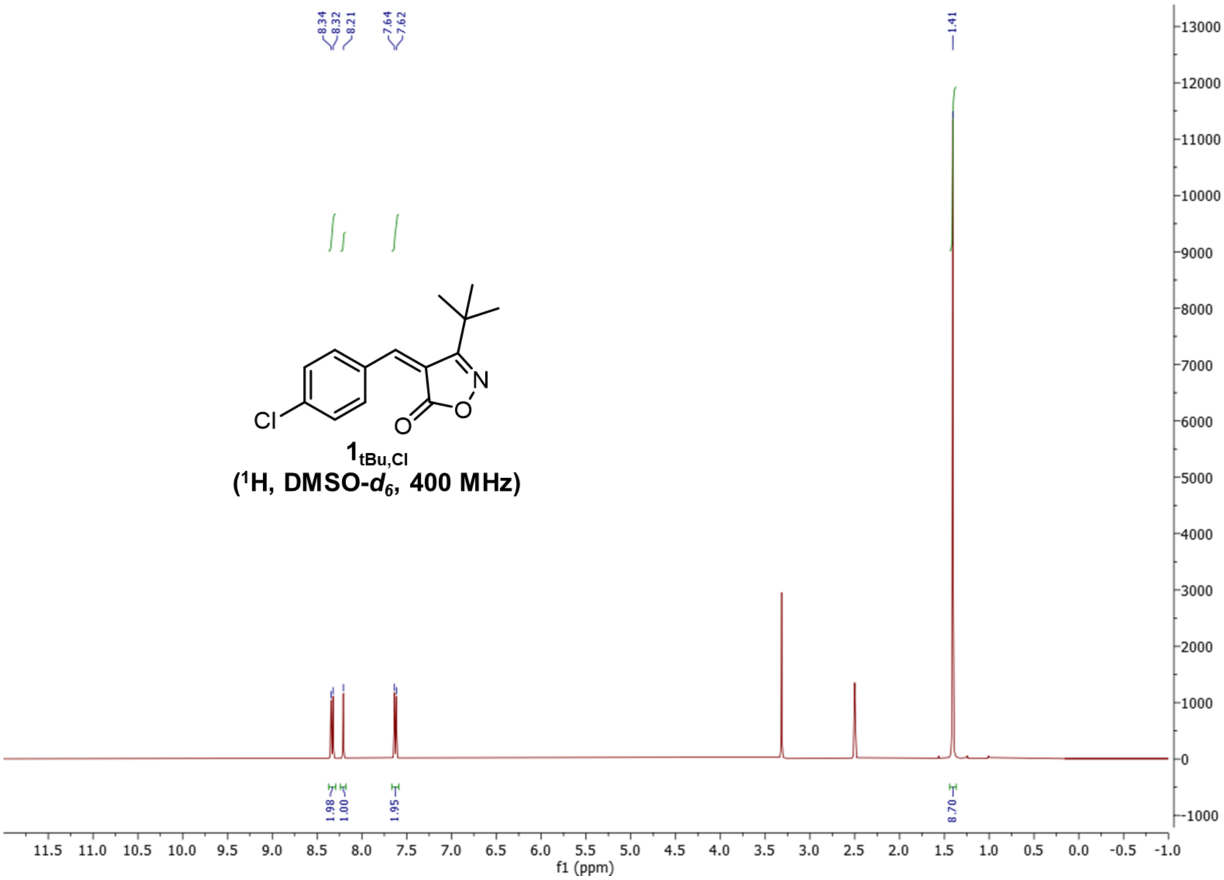
8.34
8.32
8.21
7.64
7.62
1.41
Cl
N
O
O
1tBu,Cl
(1H, DMSO-d6, 400 MHz)
1.98
1.00
1.95
8.70
f1 (ppm)

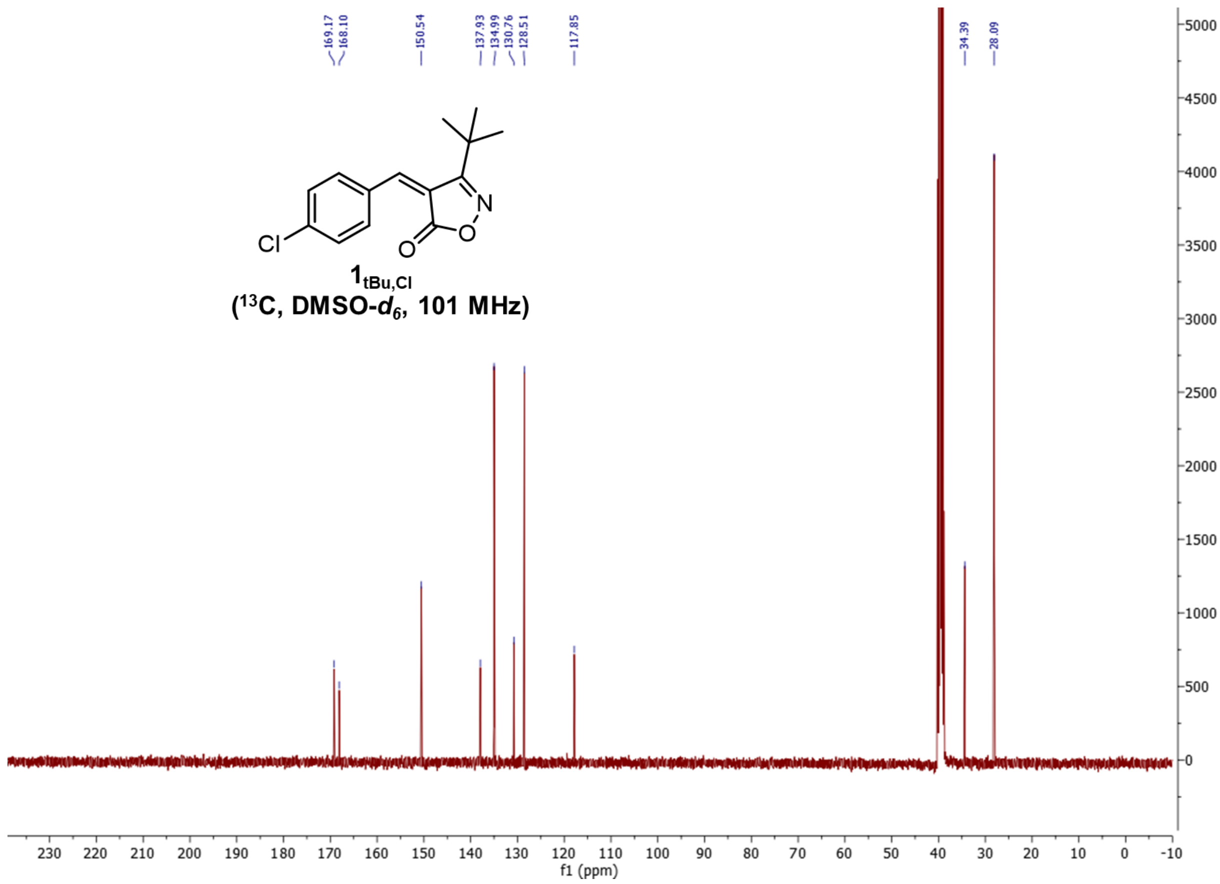
169.17
168.10
150.54
137.93
134.99
130.76
128.51
117.85
34.39
28.09
Cl
O
N
O
$1_{tBu,Cl}$
(^{13}C, DMSO-d_6, 101 MHz)
5000
4500
4000
3500
3000
2500
2000
1500
1000
500
0
230 220 210 200 190 180 170 160 150 140 130 120 110 100 90 80 70 60 50 40 30 20 10 0 -10
f1 (ppm)

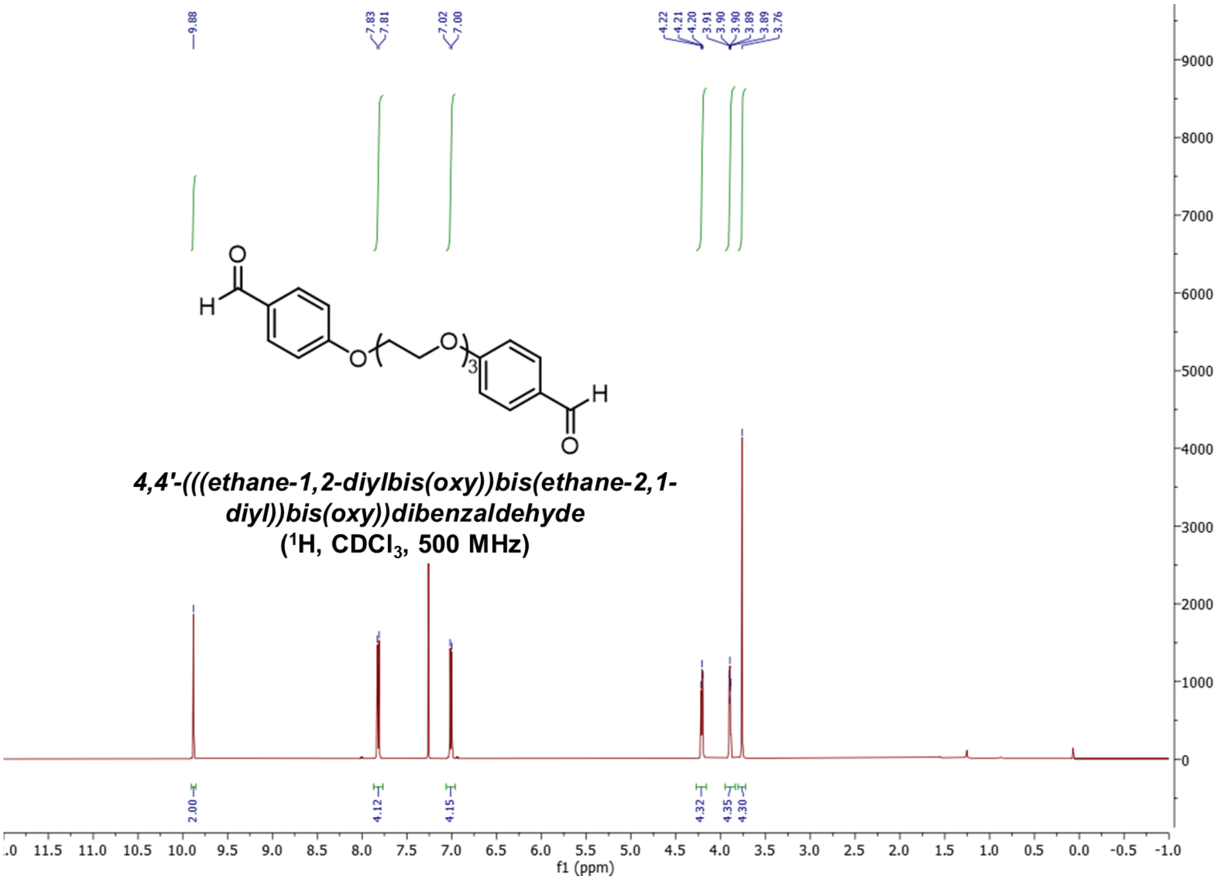

4,4'-(((ethane-1,2-diylbis(oxy))bis(ethane-2,1-diyl))bis(oxy))dibenzaldehyde
(^{1}H, $CDCl_3$, 500 MHz)

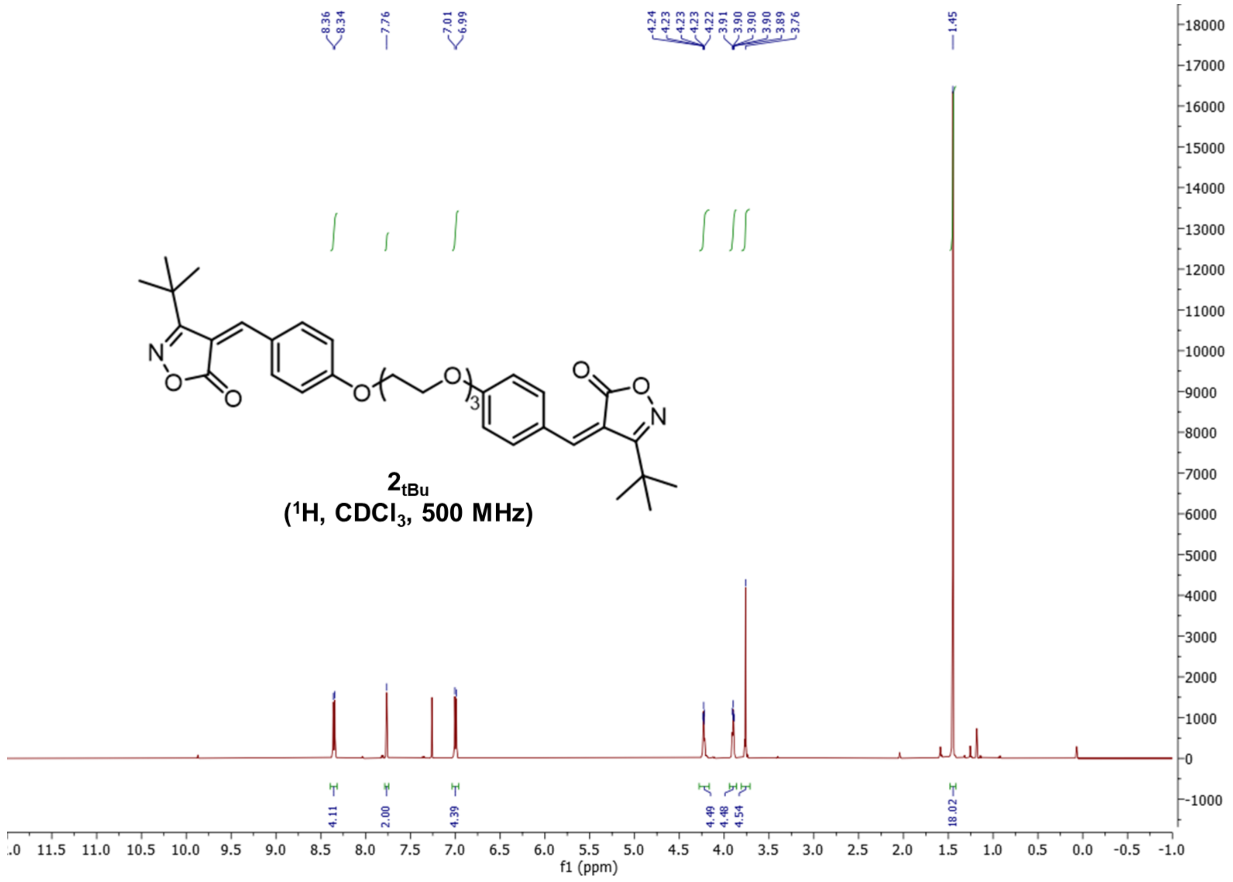

2tBu
(1H, CDCl3, 500 MHz)
f1 (ppm)

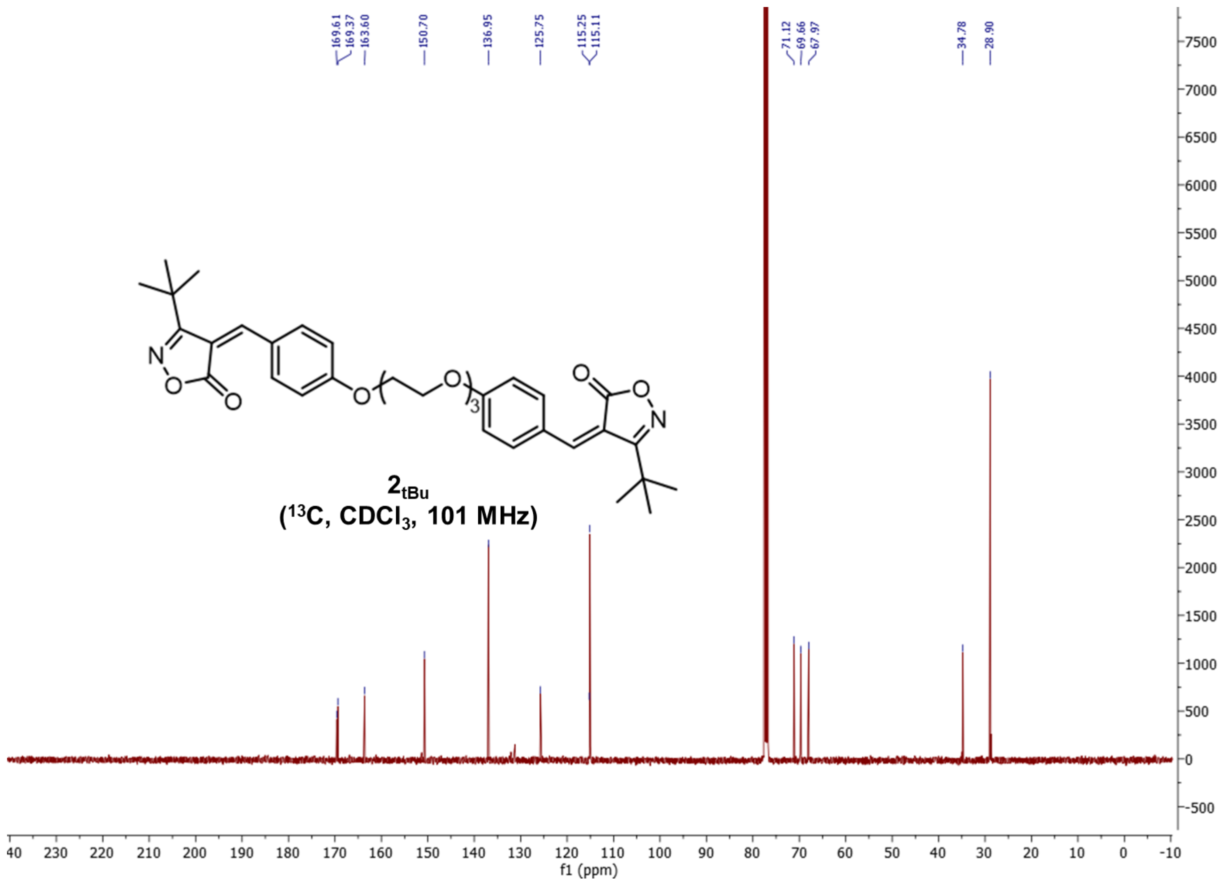

169.61
169.37
163.60
150.70
136.95
125.75
115.25
115.11
71.12
69.66
67.97
34.78
28.90
2tBu
(13C, CDCl3, 101 MHz)
f1 (ppm)
